# A multimodal spatial atlas of transcriptomic, morphological, and electrophysiological cell type densities in the mouse brain

**DOI:** 10.1371/journal.pcbi.1014106

**Published:** 2026-03-24

**Authors:** Csaba Verasztó, Yann Roussel, Lida Kanari, Sébastien Piluso, Henry Markram, Daniel Keller

**Affiliations:** 1 Blue Brain Project, École polytechnique fédérale de Lausanne (EPFL), Geneva, Switzerland; 2 Department of Mathematics, University of Oxford, Oxford, United Kingdom; 3 Open Brain Institute, Lausanne, Switzerland; University of Edinburgh, UNITED KINGDOM OF GREAT BRITAIN AND NORTHERN IRELAND

## Abstract

Brain cells can be classified according to their transcriptomic, morphological, and electrophysiological features. Comprehensive data on the spatial density of cell types that integrate all three properties are critical for improving models of how neuronal diversity contributes to brain function, yet existing atlases lack this information. To address this gap, we created a quantitative, three-dimensional atlas of cell type density distributions in the mouse brain. We began by generating a transcriptomic cell type atlas, scaling regional density estimates from brain slices using cell counts and anatomical dimensions. For densely populated regions like the cerebellum, we further refined these estimates by applying voxel-wise corrections based on average Nissl staining intensity. To connect transcriptomic identities with functional characteristics, we leveraged patch-sequencing datasets that combine single-neuron mRNA profiles, morphological reconstructions, and electrophysiological recordings from cortical neurons. Transcriptomic types were determined from gene expression data, morphological types were assigned based on structural reconstructions, and electrophysiological types were identified using K-means clustering. The resulting whole-brain atlas (consisting of 5274 transcriptomic clusters and 458 functional morphological-electrophysiological types) and computational tools offer a high-resolution (25 μm^3^ voxel size), integrative resource compatible with a broad range of neuroscience applications and enable the parsing of individual cell types to reveal previously unrecognized features.

## Author summary

This paper presents a multimodal spatial atlas of the mouse brain, integrating transcriptomic, morphological, and electrophysiological data to map cell type densities. The authors first generated a transcriptomic cell type (t-type) atlas by scaling density estimates from publicly available spatial transcriptomics datasets. For densely populated regions, they refined these estimates using voxel-wise corrections based on average Nissl staining intensity. The study then created a bridge between transcriptomic identities and functional characteristics. Using patch-sequencing data, which combines gene expression, morphological reconstructions, and electrophysiological recordings from single neurons, the researchers derived a model to convert transcriptomic types to morphological (m-types) and electrophysiological (e-types) classifications. This allowed them to create a unified 3D atlas that combines all three modalities. This resource reveals previously unrecognized features, like spatial gradients of inhibitory neurons, and enables quantitative comparisons between new transcriptomic data and older cell-counting literature. The accompanying computational tools provide a versatile platform for exploring the relationships between neuronal identity, function, and location.

## Introduction

While neural networks in invertebrates are well-mapped [1–4], the mouse brain with its ~ 70 million neurons [5] serves as a critical model for bridging simple and complex vertebrate systems. For the mouse brain, current initiatives emphasize whole-brain approaches [6–9] to decipher the vast cellular diversity within it. These and other ambitious efforts [10–12] have yielded insights into the brain's complex organization and function. Over the past decade, single-cell RNA sequencing (scRNA-seq) [13–15] and single-nucleus RNA sequencing [16–18] revealed how gene expression varies across different brain regions [19–21]. Recently, comprehensive sampling of the entire mouse brain, often with single-molecule precision resolution [22,23] and within a common platform [24], now allows high-resolution mapping of neural cell types across the brain [25, 26]. The integration of large amounts of spatial transcriptomic data [9,27] in the whole-brain broader context revealed complex interregional relationships, and precise analysis of tissue characteristics. However, since no mouse brain has yet been fully mapped at the single-cell level, estimations of transcriptomic cell type placement will remain a necessary tool for researchers until complete cellular identification can be realized on the whole-brain or whole-body scale.

Using transcriptomic data [8,9] and the common coordinate framework from the Allen Institute for Brain Science (AIBS) [27], we created 3D atlases that map the spatial distribution of distinct transcriptomic cell types (t-types). We extracted cell coordinates and their brain region’s volume from aligned brain sections to estimate cell densities across all brain regions. As sections often provide multiple samples from a region, coverage of the mouse brain is well-validated within the constraints of this methodology and almost complete.

Additionally, we developed a method for converting between various cell type classification systems. Recent advances in patch-sequencing technologies link transcriptomic profiles of individual cells with morphological types (m-types), which categorize cell shape and structure, and electrophysiological types (e-types), which categorize cells according to electrophysiological behavior [28–30]. From such experimental data, we derived a model to convert between various cell type classifications and recreate the 3D atlas for all other functional m- and e-type classifications. Aligning transcriptomic, morphological and electrophysiological modalities and validating putative correspondences remains challenging because each modality has different sampling biases, measurement noise and metadata conventions. Community portals and Python-based reproducible workflow frameworks that promote standardized formats, provenance tracking and shared analysis pipelines (e.g., the neocortical microcircuit portal [31,32] and the broader Python/NWB/DataJoint [33] ecosystem) have proven useful for mitigating these issues and enabling cross-modal validation. We therefore built our conversion and atlas pipeline with these principles in mind to improve reproducibility and to make cross-modal comparisons more robust.

Our atlas revealed gradients and partial intermixing of certain inhibitory neuron types across cortical and subcortical regions. These patterns were not obvious from prior single-modality datasets and suggest that inhibitory circuits may be more spatially nuanced than previously appreciated. By integrating Patch-Seq data, we demonstrated that cell-type morphological and electrophysiological traits correlate with their transcriptomic identities in a spatially structured manner. This provides a more complete view of how neuronal identity, function, and location are interlinked in the brain. Previous scientific effort has focused on mapping the densities of inhibitory and excitatory neurons in the brain. The methods developed in this paper go beyond this by integrating cell type modalities (t-, m- and e-) on a whole-brain scale. Converting transcriptomic cell clusters into previous functional morphological-electrophysiological type classifications (me-classifications) bridges the gap with classifications present in the traditional body of literature, allowing quantitative comparison of older cell counting results with transcriptomic results. This provides neuroscientists with an accurate cell density map of the mouse brain that they can easily interpret and model, for example in large-scale simulators that consume atlas-based densities [34,35]. In addition, the accompanying computational framework allows researchers to dynamically query, compare, and model cell-type densities, providing a versatile platform for uncovering previously hidden structure-function relationships (e.g., morphological and electrophysiological profiles correlate strongly with known anatomical hierarchies, unexpected gradients and intermixing of certain inhibitory cell types across cortical and subcortical regions / discovery of novel spatial gradients and transitions, statistical relationships between cell-type distributions). Of note, these tools enable the testing of targeted hypotheses on why certain brain areas are affected in neurological disorders and to develop more accurate, biologically grounded models of circuit-level behavior. Last, the integration of multimodal data, voxel-level density mapping, Patch‑Seq alignment, computational modeling tools, and data harmonization methods collectively provide a new approach for comparative and cross-species neuroscience studies.

## Methods

### Atlas construction: whole-brain, voxel-scale cell-type density atlas

#### Data used for models.

**Fig 1:**
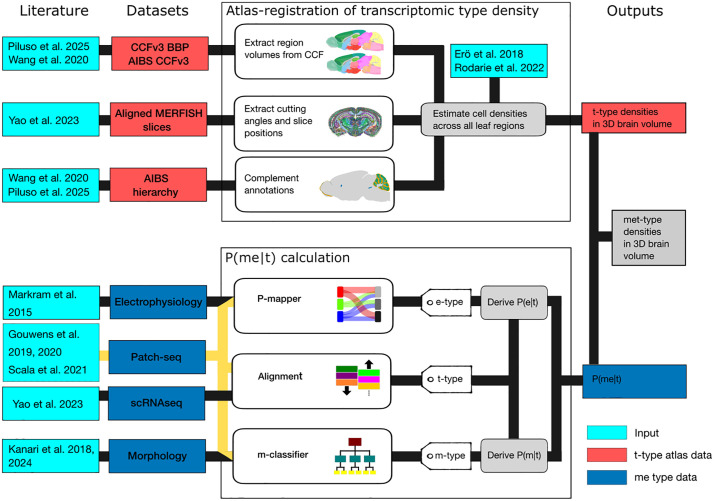
Pipeline overview from 3D mapping to linking cell type identities. The top module shows the workflow for building 3D transcriptomic cell type atlases from MERFISH data. We produced both unscaled and scaled versions of the densities by (i) integrating various datasets with MERFISH metadata, (ii) calculating cell type densities for each leaf region of the mouse brain, and (iii) projecting these densities back into the average brain space. T-type density estimates can subsequently be integrated with other neuron phenotypes through a probabilistic mapping framework. The bottom module derives P(me−type∣t−type) from patch-seq data. The reference dataset for the three modalities was used to align the patch-seq datasets on established classification and derive the probability of observing an me-type given a t-type. The P(me−type∣t−type) can be combined with the t-type density atlas to produce an met-type density atlas. The workflow integrates all these datasets and literature values and no additional input is required.

In this study, we combined several publicly available whole-brain datasets to provide a more robust and detailed perspective: The Allen Brain Cell (ABC) Atlas [24], the multiplexed error-robust fluorescence *in situ* hybridization (MERFISH) dataset [19,24], the extended and improved version of the latest common coordinate framework annotation (CCFv3) and Nissl volume [36] from the AIBS and the Blue Brain Project, and two patch-seq datasets [29,30] (Fig 1). We recommend reviewing the referenced resources and methodologies beforehand, as they may help contextualize and enhance understanding of our approach.

The ABC Atlas is an online platform that provides a comprehensive cell type atlas, visualizing transcriptomic cell type expression, spatial organization, and diversity across the brain. MERFISH is a high-resolution spatial transcriptomics technique that quantifies and maps RNA molecules within cells. For our analysis, we downloaded both the complete mouse brain taxonomy (hierarchical clustering) and MERFISH dataset from the platform. All analyses were conducted using the 2024-03-30 release of the ABC Atlas to ensure consistency and alignment with the latest available data. Patch-seq datasets are valuable because they link RNA sequencing, morphological reconstruction, and electrophysiological recordings for the same neuron type. Each used dataset focuses on a different region of the mouse cortex: primary visual cortex for [29] (~500 inhibitory neurons) and primary motor cortex for [30] (~200 pyramidal cells and ~300 inhibitory neurons).

#### Extending CCFv3’s hierarchical annotations and parcellations for comprehensive dataset integration.

To integrate these heterogeneous datasets into a unified spatial framework, we next harmonized and extended the anatomical hierarchy of the common coordinate framework. First, we extended the hierarchical annotation of the AIBS CCFv3 annotation volume [27] for the whole dataset allowing all voxels to be assigned to a leaf region (the term *leaf region* represents the deepest level of the mouse brain ontology). We also extended the parcellation to parcellation term membership metadata that was created for the ABC Atlas (Fig 1 *top-right*). These steps were necessary to resolve inconsistencies between the original structural annotations of the CCF and the ABC Atlas parcellation system. We incorporated label IDs and alternative cluster names, omitting special symbols to facilitate easier processing. Finally, the *main olfactory bulb, glomerular laye*r was added to the list as a substructure, as well as all granular, molecular, and Purkinje layers of all the lobules of the cerebellum. In all steps, we adhered to the ABC Atlas’s new multimodal reference system which introduced parcellations, while maintaining backward compatibility with the CCFv3’s hierarchical annotations.

#### Aligning and resampling brain annotation volumes for improved cell density estimation.

Following the extension of the anatomical hierarchy, precise spatial alignment between MERFISH data and the reference annotation volumes was required to enable accurate volumetric density estimation. The ABC Atlas provides an aligned and resampled annotation volume in which region IDs were renumbered for better visualization. However, it is not perfectly aligned with the CCFv3 annotation volume. Therefore, we used the original CCFv3 annotation volume (at 10 μm^3^ voxel resolution) and identified all original region IDs by matching voxel counts in the corresponding annotation volume slice for every cell. This gave access to a rotated, resampled and aligned annotation array with exact volumes for every brain region intersected by MERFISH slices (Fig 1 *top-center*). For example, a representative slice intersected a large number of voxels from leaf region 1, a moderate number from region 2, and a small number from region 3, illustrating how regional volumes were quantified across slices. Consequently, cells of every cell type located in these brain regions acquired the exact brain volume of their brain region within the brain slices as metadata. Acquiring this information allowed us to overcome issues like estimating cell type densities in multiple patches (e.g., a brain region was intersected in both hemispheres by the same MERFISH slice), or estimating cell densities by triangulating region volumes around their CCF coordinates.

#### Estimation of cutting angles and coronal region volumes for MERFISH sections.

For the coronal MERFISH sections, cutting angles (tilted in both horizontal and vertical directions) and coronal positions were estimated using normalized-mutual information (NMI) metrics [37], aided by the provided average template array (Fig 1 *top-center*). For all 53 coronal slices the estimated axial angles were 36.84 ± 3.3 degrees, while the vertical angles were 6.36 ± 0.27 degrees (where 0° would indicate no tilt in alignment). This information was used to calculate the region’s volume of the MERFISH section in the plane for each individual cell.

For every cell in the ABC Atlas database next to its single reconstructed z coordinate (i.e., along the coronal axis), we added their voxel position, their template number, cutting angles, the region ID of their leaf region, together with the region’s voxel count (i.e., voxelized volume) (Fig 1 *top*). Cells which did not pass the ABC Atlas quality control (QC) were not included in the calculations. Additionally, 15 cells were removed from the database because their incorrect x_ccf and y_ccf coordinates were 0. Although these cells passed the ABC Atlas QC, we could not use them since they were physically located outside of the brain.

It is important to note that MERFISH slices did not completely cover every brain region: e.g., the *frontal pole, layer 1*, and retrosplenial area, dorsal part, layer 4 were not covered in this dataset. We included S1 Table listing all leaf regions with no direct coverage. Two substructures covering white matter of the brain were not broken down to their respective leaf regions in the AIBS’s parcellation annotations; thus they were used instead, which gives these areas a coarser resolution. These regions are: *corpus callosum*, *anterior forceps* with two regions, the inferior *cerebellar peduncle* with two regions, the *sensory root of the trigeminal nerve* with two regions, the *superior cerebellar peduncles* with four regions, and the *stria terminalis* with two regions. These regions inherited the same set of cell types and densities. We also recognized that zero density values can convey valuable biological information; thus, for regions without assigned voxels in the 3D reference space, we used N/A (not a number) to distinguish between the lack of information and the absence of cells.

Further, artifacts from imperfect sectioning, tissue damage, or chemical processing of the slices could obscure the estimated volumes. However, many of these irregularities could be resolved or corrected during the alignment of MERFISH sections to the common coordinate framework. We estimated volumes from the annotation volume file registered to the MERFISH slices and incorporated this data into the metadata for each cell. Missing tissue, bad quality cell information or cells registered to uncharacterised regions could not be recovered.

After the preceding steps, 3,791,571 cells remained. These cells belonged to 677 regions in a total of 53 coronal sections. Out of these regions, the newly added main olfactory bulb (MOB) layer and the cerebellar lobule layers remained empty, as they were either not part of the ABC’s parcellation annotations or no cells were identified in the region which passed QC. We split these substructures into their respective leaf regions based on a simple set of rules. Cerebellar lobules can be split into Purkinje, molecular, and granular layers. Purkinje layers inherited 100% of the Purkinje cells, 100% of the Bergman glia, and they shared microglia equally with the other two leaf regions. Molecular layers were set to be purely inhibitory, thus they do not contain any Bergmann glia, Purkinje cells or cells expressing the neurotransmitter glutamate (Glut). Granular layers inherit every cell type, except Purkinje cells and Bergmann glia; and only contain Golgi cells from the gamma-aminobutyric acid (GABA) expressing cell types. On the other hand, the missing *main olfactory bulb, glomerular laye*r inherited all the cells from the unassigned region of the MOB.

Cells which were assigned to unassigned brain regions in the ABC Atlas were omitted from the calculations. This omission did not influence regional cell densities but reduced somewhat the overall cell count. Note that we have only included cells from the ABC Atlas where the CCF positional coordinates were identified, as the alignment process of MERFISH sections was not available to us. To reduce data processing and avoid potential signal spillover of cells from neighboring regions due to misalignment, we decided to not include sections which were not aligned to the common coordinate framework. Instead we rigorously tested the ABC Atlas alignment ourselves to check for misalignment which could be detected in regions where cell composition was known from the literature.

#### Region structuring in the density atlas.

To work out a widely usable density atlas we adapted the parcellation annotation with 4–5 levels of hierarchy. To make it compatible with the original brain hierarchy used by older versions of the anatomical annotation volumes, we introduced missing leaf regions as a substructure. We handled gray and white matter regions in a similar manner. In the case of gray matter, we split those substructures which could be further split into leaf regions (the term *leaf region* represents the deepest level of the hierarchical ontology). For white matter, we initially treated parent regions undivided, assuming homogeneity. Later, their leaf regions were assigned the same density information as their parent regions. This provides the flexibility to select any brain region at any hierarchical level by concatenating its substructures. As a result, brain volumes identified in the MERFISH slices can be translated one-to-one to every level of hierarchy in the mouse brain anatomical reference atlas.

#### 3D cell density maps from MERFISH data.

**Fig 2:**
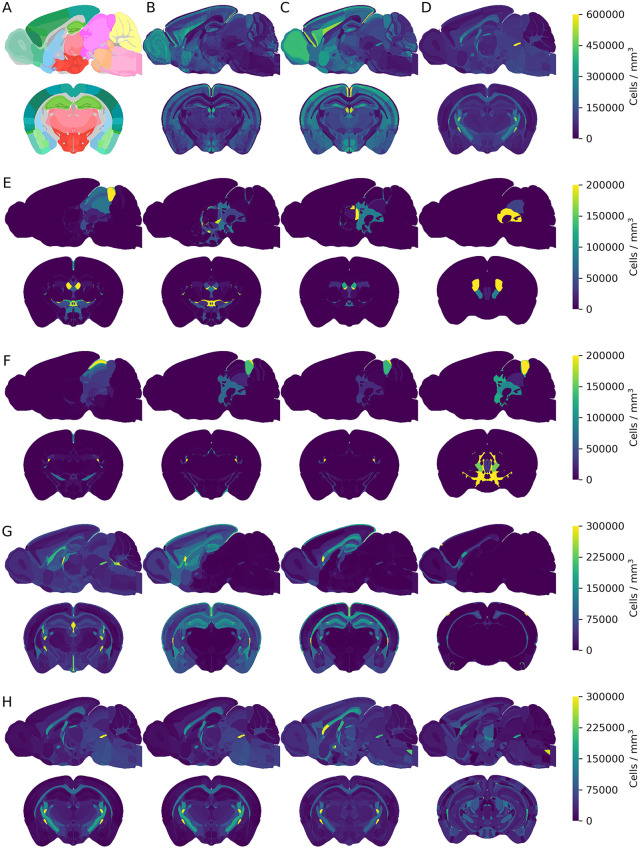
Regional transcriptomic cell counts in the extended 3D atlas format (566, 320, 456). Sagittal (y = 200) and coronal (x = 300) sections from the **A.** extended and improved CCFv3 annotation volume (The annotation colors match those in the AIBS reference atlas.), **B.** regional cell counts with Nissl granularity, **C.** regional neuron counts (averages), and **D.** regional non-neuronal counts (averages). Colorbar shows cell numbers in number of cells / mm^3^ for panel B. **E.** Sagittal (y = 200) and coronal (x = 300) sections of an excitatory cell type at 4 different hierarchies (class, subclass, supertype, cluster). **F**. Inhibitory cell type examples at different hierarchical levels. **G.** Astrocytic cell type examples at different hierarchical levels. **H.** Oligodendrocytic cell type examples at different hierarchical levels. Colorbars (**E-H**) show cell numbers for the first cell type in the row. Resolution: 25 μm^3^ voxel size.

Processing all 53 MERFISH slices yielded 5,274 individual 3D brain volumes (for each cell type cluster) within the ABC Atlas. Densities were estimated for each cell type in each region by dividing the total number of cells in that region across all aligned slices by the sum of the corresponding region volumes. Additionally, we grouped cell types based on taxonomic hierarchy, creating 3D density volumes for major cell groups such as excitatory neurons, inhibitory neurons, modulatory neurons, glial cells, and all non-neuronal cells, among others (Fig 2). As a result, in each brain volume, leaf regions contain the estimated average cell density of the specified cell type, including t-type clusters or larger cell groups, calculated from MERFISH slices that intersected the region. For normalization and validation, we also generated brain volumes where voxels represent total cell counts in each region for the major cell groups. To reduce file numbers, we calculated cumulative densities of major cell groups at each level of the anatomical hierarchy, allowing them to be stored efficiently in a single table.

#### Cell Density Estimation and Scaling Across Brain Regions.

We estimated cell type densities in brain regions where alignment to the common coordinate framework was available by calculating region volumes from the voxelized region size per slice multiplied by section thickness (10 µm in the fresh-frozen state), and summing all cells per cell type divided by the region’s total volume across all intersecting slices (Fig 2). The extended and enhanced CCFv3 annotation volume provided a straightforward way to visualize cell densities (Fig 2A), and the ABC Atlas transcriptomic taxonomy tree enabled aggregation of cell type clusters into broader categories such as excitatory and inhibitory neurons, astrocytes, or oligodendrocytes (Fig 2E-2H). Pooling average densities across the brain allowed characterization of general populations, including all cells and neuronal versus non-neuronal groups (Fig 2B-2D). MERFISH slices covered 565 parcellation substructures with cell types passing QC, which, after division into leaf regions following the annotation hierarchy, resulted in cell coverage for 670 of the 677 possible regions. Of the seven missing regions (*frontal pole, layer 1, central canal, spinal cord/medulla, pyramidal decussation, bed nucleus of the anterior commissure, cuneate fascicle, vomeronasal nerve, Gracile nucleus)*, only the frontal pole belonged to gray matter. For these regions, cell type numbers were reported as N/A, although approximate densities were estimated using scaling methods; more accurate estimates could be achieved by incorporating additional MERFISH slices. Using voxel-based volumetric information, we calculated total cell numbers for every cell type or group and refined initial whole-brain density estimates by scaling them to align with reference data reported in the literature [7] using three methods: global scaling, cell type density transplant, and Nissl staining based estimation (S5 Fig), which can be selected according to use case. In the global scaling method, all 5,274 MERFISH cell types were pooled into hierarchical groups based on the ABC Atlas taxonomy, separating neurons from non-neuronal classes. Neuronal classes included 29 groups, comprising 15 excitatory, 12 inhibitory, and two modulatory classes identified by their primary neurotransmitter. Astrocytic, oligodendrocytic, and microglial clusters were grouped as glia, while remaining non-neuronal cell types were classified as other non-neuronal cells. Neuron scaling rules for primate brains [5] were applied to set upper limits of 12,618,420 neurons in the cerebral cortex, 41,825,100 neurons in the cerebellum, and 16,446,480 neurons in the rest of the brain, preserving relative differences between regions and cell type groups. An exception occurred in the cerebellum, where the ABC Atlas does not distinguish granular, molecular, and Purkinje layers; subdivision of lobules into leaf regions enabled separate scaling of excitatory neurons in granular layers while keeping inhibitory neuron numbers constant, and scaling non-neuronal cell types across all layers. In the cell type density transplant method, peer-reviewed literature values or user-provided inputs [7] served as ground truth for selected regions by directly editing the density values of corresponding clusters. For global scaling to a final total of 70.89 million neurons, regions with transplanted densities were kept constant, and scaling from the first method was applied only to non-transplanted regions. A third scaling method was based on the average Nissl template, in which densities were scaled to reflect Nissl intensity values in each brain region. The template was generated from 86,901 coronal Nissl-stained slices from 734 postnatal day 56 C57BL/6J mouse brains from the AIBS *in situ* hybridization data portal [36], mapped to a 10 µm³ isotropic volume and normalized by the number of intersecting sections. Scaling was performed either by assigning a specific density to a reference intensity value, such as the highest average intensity in the *crus 2 lobule granular layer* set to 4 million cells/mm³, or by assigning the lowest average intensity, observed in the lateral preoptic area, to its corresponding density, with proportional scaling applied across regions (S6 Fig). Within each region, relative cell type ratios determined final densities. Although the relationship between Nissl staining intensity and cell density is not strictly linear due to differences in cell size, cell type composition, and RNA content [38,39], Nissl intensity provided a useful proxy for relative cell density across brain regions under controlled conditions.

### Normalization strategies, estimate adjustments

#### Adjustments for regions with high cell density.

To address the limitations of MERFISH-based estimations, we could enhance its accuracy by scaling the density values (S7 Fig). However, the challenge with scaling was that it relies on the adoption of a scientifically reviewed experiment from the literature as the reference point. Therefore, we proposed offering multiple methods for scaling density values across the brain to accommodate different use cases and expectations.

The first scaling method involves aligning the global values with those obtained through isotropic fractionation, specifically the neuron scaling rules for primate brains. This approach allowed us to scale the total counts of neurons to 70.89 million neurons and the total cell counts (glia included) to 108.69 million cells across every brain region. This scaling approach allowed us to adjust cell numbers across large brain regions while preserving the relative densities between regions and cell types. However, a limitation is that certain regions may retain errors and stand out as outliers due to issues such as poor coverage, low-quality slices, or compromised image quality.

In the second scaling technique we wanted to introduce density transplantation. For example, specific modeling studies require a defined set of neuron types, including particular density and standard deviation, often due to computational limitations or measurements that are not publicly available. In our scaling pipeline we provided the ability to swap out the estimates from MERFISH values with specific values for any given cell type or cell class (or at any level of the cell type hierarchy) while keeping the other regions constant. In this case, we can still apply the first scaling method while the pipeline will take into consideration that transplanted regions have to stay constant in any subsequent changes. Since in the first step we need to scale up or down the total number of cells across regions to meet a maximum number (e.g., 70.89 million neurons or 108.69 million with all cells included) the neighboring regions in the brain area will get scaled up or down depending on the transplanted values. In this study we transplanted 51 regions (including larger regions, e.g., *Striatum*) where we had estimates of total neuron or inhibitory and/or excitatory cell densities [38]. After transplantation we ran our first global scaling method based on the neuron scaling rules for primate brains while keeping those densities constant which were fixed by the transplant operation.

The third method, average Nissl volume based scaling, also provided a way to scale density values without changing the ratio of individual cell types (S6 Fig). It also enabled us to estimate cell densities in regions that were not covered by the MERFISH slices.

#### Voxel-level granularity using Nissl intensity.

As cell type densities are calculated for each leaf region, densities remain constant within each region and do not vary on a voxel-by-voxel basis across the brain, except at regional borders. To address this limitation, we used the average Nissl template derived from 734 Nissl-stained mouse brains [36] and calculated voxel-level granularity within each brain region by normalizing Nissl intensity values relative to the mean. These normalized voxel values were then applied to each cell density in the 3D representation. We chose this template due to its alignment with the extended and improved CCFv3 annotation volume, though other Nissl-stained slices can also be used (S4 Fig). This adjustment replaces the constant average values of a given cell type with values that better reflect spatial variability. This step is optional, but one limitation to consider is that each cell type is normalized with the same Nissl-derived factor across its region, even though Nissl intensity represents the cumulative signal of all cell types in a given voxel, regardless of their specific contributions or ratios in that area. This approach maintains the region's overall density by keeping the cell type ratios constant; however, we lack information on the true ratio of cell types from one voxel to another.

In summary, our pipeline provides the ability of scaling or transplanting densities across the whole brain with voxel level granularity while leveraging the benefits of the transcriptomic atlas’s cell type resolution. At this stage of the pipeline, various types of biologically relevant experimental data can be incorporated into the 3D atlas and converted into transcriptomic densities. The pipeline up to this stage is publicly available at https://github.com/BlueBrain/Molsys-transcriptomic-atlas, and further example code and tutorials are provided at https://github.com/cveraszto/TME-types.

### Alignment of transcriptomic cell types with morphology and electrophysiology

#### Alignment of mRNA to established neuronal t-types.

To link transcriptomic cell-type densities with functional neuronal properties, we aligned transcriptomic types to morphological and electrophysiological classifications derived from patch-seq data. One of the first steps of the pipeline was to assign reference t-types defined by [8] in their scRNA-seq study to patch-seq datasets using mRNA data (Fig 1 *bottom panel*). We used the trimmed means (25% - 75%) of the scRNAseq dataset as reference points for the t-types alignments. To process and align gene expression profiles we integrated several libraries, including *scanpy*, *mygene*, and *UMAP* [39, 40]. We first cleaned the t-types from Yao et al. [8] using a reproducible preprocessing pipeline implemented in Python. Briefly, gene identifiers in the Yao reference dataset were mapped from Ensembl IDs to gene symbols with the *mygene* API [41]. Only genes that appeared exactly once in the Yao reference table were retained to build the *unique_genes* list. For the patch-seq expression data we excluded samples annotated as *“low quality”* and we summed exon and intron counts per cell for the filtered Scala cell list. We performed data-driven gene selection using an established routine [30] on raw counts. The routine selects genes by comparing the frequency of zero (or near-zero) expression to mean log₂ nonzero expression and returns a selection of genes with high frequency of zero expression and high level of nonzero expression by fitting an exponential decay (parameters *yoffset = 0.04*, *xoffset = 6.2* and *decay = 1.2*). To align datasets we restricted the Yao matrix to the *unique_genes list*, took the intersection of gene sets between the reference and sample, and constructed *scanpy’s AnnData* objects for MERFISH (Yao) and Scala (patch-seq) using these common genes. We computed PCA, neighborhood graph and UMAP on the MERFISH reference (*sc.pp.pca*, *sc.pp.neighbors*, *sc.tl.umap*) and then ingested the Scala samples into the MERFISH embedding (*sc.tl.ingest*). For label transfer we assigned to each Scala cell the nearest MERFISH t-type in PCA space by Euclidean distance. The resulting alignment table was post-processed to extract hierarchical Yao reference labels and Scala family/type annotations. Finally, when building contingency maps between old (Yao) and new (Scala) t-types we restricted downstream comparisons to mappings supported by at least three cells of a given type. The exact parameter values and the code used to generate all intermediate files (including *Scala_aligned_labels.csv*) are provided in the Supplementary Code repository (https://github.com/YannRoussel/probabilistic_mapping_extension) to ensure full reproducibility.

To visualize the alignment results, we generated heatmaps showing the distribution of patch-seq cells relative to reference t-types. The cells were further divided into excitatory and inhibitory subtypes to enhance interpretability. We used this data to illustrate the alignment fidelity for both excitatory and inhibitory cell populations (S1 Fig).

#### Morphological clustering.

Morphological clustering was conducted using neurons from various brain regions with data obtained from the Blue Brain dataset [31,32], combined with patch-seq data from [29] and [30]. Morphological reconstructions were processed with the *morphology-workflows* package to curate and align the different datasets [42]. Subsequently, the topological descriptors were extracted from the morphological reconstructions using the *Topological Morphology Descriptor (TMD)* package [43]. *TMD* provides a topological description of neuron morphologies, capturing key characteristics of neuronal morphologies that can be used for classification and clustering of neurons [44]. Each neuronal tree is represented as a persistence barcode, which tracks the start and the end radial distance of each branch in the underlying tree structure. From each neuronal reconstruction two persistence images [45] (cite: Adams et al) were generated based on (1) the topological barcode of the axonal branches and (2) the topological barcode of the dendritic branches. Pyramidal cells were processed independently from the interneurons, according to the expert labeling, due to the significant difference in dendritic shapes.

The persistence images were vectorized and used as input to an unsupervised clustering algorithm (K-means). The number of clusters were selected based on the optimal number of clusters that approximated the Blue Brain dataset that were already classified from experts [32,46], which yielded optimal results for 10 clusters of axonal and 10 clusters of dendritic shapes. Then, all the datasets Blue Brain dataset [32], combined with patch-seq data from [29] and [30] were clustered based on the same clustering algorithm optimized to distinguish the classes in the Blue Brain dataset. Through this process each neuronal morphology was assigned a DendriticCluster and an AxonalCluster, the group was selected from 10 different groups according to the unsupervised K-means clustering algorithm.

Canonical m-types were named following a standardized convention to reflect both neuron main type and clustering results, formatted as follows: NeuronType_DendriticCluster_AxonalCluster (e.g., IN_DEND_0_AX_2 for inhibitory neurons with dendritic cluster 0 and axonic cluster 2). This schema resulted in 200 potential m-types, derived from the combination of 2 neuron types, 10 dendritic clusters, and 10 axonic clusters. However, fewer m-types are observed in practice for the patch-seq data of [30] and [29], as not all dendritic and axonal cluster combinations are present in the datasets (see supplementary m-type list). Morphologies from patch-seq datasets were classified into m-types based on their projected location in the morphological feature space. The probability of observing a specific m-type given a t-type was derived by analyzing their occurrences in the patch-seq datasets. We defined:


$$\begin{array}{c} P({m-type}_{i}|{t-type}_{j})=\mathrm{card}({m-type}_{i}\cap {t-type}_{j})/\mathrm{card}({t-type}_{j}) \end{array}$$

Where card(x) is the cardinal (i.e., the number of elements) of the set x.

#### Electrophysiological classification.

To compute the conditional probabilities of observing specific e-types based on t-types, we used data from a reference dataset [35] alongside the patch-seq datasets. First, electrophysiological features (e-features) were extracted from recordings using the *EFEL* package, following established protocols [47]. To standardize the data and mitigate variability induced by input resistance, traces were relabeled as a percentage of the rheobase current. For classification, we focused on features extracted from traces presenting rheobase percentage available across all cells. Data cleaning was performed to retain only the shared feature columns, eliminating rows and columns with missing values to ensure consistency across datasets. Each e-feature dataset was subsequently standardized with *StandardScaler* from *scikit-learn (1.4.2)* [48].

To cluster these features, we then used hierarchical clustering (via *AgglomerativeClustering* from *scikit-learn (1.4.2)*) to assign each sample to one of 20 feature-based clusters common to the patch-seq and reference datasets. Selected features were prioritized for their relevance to electrophysiological behavior while maintaining independence from t-type labels, thus capturing essential biological variation across cell types. We used previously published data as a reference dataset for e-types that offers a collection of traces with reference labels from the Petilla convention (e.g., cNAC, [35]). The obtained clusters helped structure the data for probabilistic mapping. The probabilities of observing an e-type given a t-type were computing as done previously [49] according to the following equation:


$$\begin{array}{c} P({\text{e\textbackslash{} -\textbackslash{} type}}_{i}{\text{|t\textbackslash{} -\textbackslash{} type}}_{j})=\sum_{k=0}^{N}P({\text{e\textbackslash{} -\textbackslash{} type}}_{i}|C_{k})\cdot P(C_{k}|{\text{t\textbackslash{} -\textbackslash{} type}}_{j}) \end{array}$$

with

• $e-type_{i}$ the ensemble of elements with the *i*^*th*^ e-type in the reference dataset (SSCx).

• $t-type_{j}$ the ensemble of elements with the *j*^*th*^ t-type in the patch-seq datasets.

• N the number of common clusters.

• $C_{k}$ the *k*^*th*^ cluster.

• $N_{e-types}$ the number of e-types.

• $N_{t-types}$ the number of t-types.

• $\begin{array}{c} P({\text{e-type}}_{i}|C_{k})=\frac{\mathrm{card}({\text{e\textbackslash{} -\textbackslash{} type}}_{i}\cap C_{k})}{\mathrm{card}(\overset{{\text{N}}_{\text{e-types}}}{\underset{m}{\cup }}{\text{ e-type}}_{m}\cap C_{k})} \end{array}$

• $\begin{array}{c} P(C_{k}|\vee {t-type}_{j})=\frac{card({t\;-\;type}_{j}\cap C_{k})}{card({t\;-\;type}_{j})} \end{array}$

To further analyze the correspondences between t-types and e-types, a probabilistic mapping matrix was computed, showing the conditional probabilities for each t-type/e-type pair. This matrix was visualized as a heatmap (S1 Fig), illustrating the probability of observing particular e-features given the assigned t-type. Additional post-processing on specific labels was carried out to adjust for excitatory (Glut) and inhibitory (GABA) cell classifications.

#### Probabilistic mapping and extrapolation.

To compute the joint probabilities of observing combined morphological-electrophysiological types (me-types) given transcriptomic types, we utilized two initial probability matrices: P(m−type∣t−type), representing the probability of observing a m-type given a t-type, P(e−type∣t−type) representing the probability of observing an e-type given a t-type. We combined these matrices under the assumption that m-type and e-type classifications are independent of each other, allowing us to approximate the joint probability P(me−type∣t−type) by the product P(m−type∣t−type)×P(e−type∣t−type).

In the implementation, we assumed that e-types and m-types are conditionally independent given a t-type. Under this assumption, the joint probability P(me−type∣t−type) can be approximated by the product $\begin{array}{c} P(m-type|t-type)\times P(e-typ|\vee t-type) \end{array}$. While this is a strong assumption, it was inevitable given the way the two distributions were obtained: P(m−type∣t−type) was derived from direct predictions on individual neurons with known morphologies, whereas P(e−type∣t−type) was estimated using a previously established probabilistic mapping approach [49]. Direct prediction of e-types at the single-cell level was not feasible due to the nature of the labels, which were assigned by experts rather than being directly observable. To approximate joint me-type probabilities, each column of the P(e−type∣t−type) matrix (i.e., each e-type) was used to scale the P(m−type∣t−type) matrix element-wise. This was accomplished by iterating through the columns of P(e−type∣t−type), multiplying P(m−type∣t−type) matrix by the corresponding e-type probabilities for each t-type. The resulting matrices were then assigned composite column labels reflecting the combined me-type identity (e.g., m−type∣e−type). This approach yields a final reduced probabilistic map P(me−type∣t−type) that approximates the joint distribution of me-types for each t-type as a first-order estimate. The validity of the independence assumption and its potential impact on the results is provided in the Discussion section.

To extrapolate me-types for t-types not represented in the patch-seq datasets, we implemented a multi-step approach. Initially, we identified gene markers pertinent to the electrophysiological and morphological properties of neuronal types by applying feature selection methods across various gene expression profiles. RNA-seq data from both covered and uncovered t-types were preprocessed, and feature selection for e-features was conducted using MultiTaskLassoCV (cross-validation) and Random Forest regression models, while morphological features (i.e., m-types) were analyzed using Logistic Regression with cross-validation, Random Forest classifiers, and mutual information classification from *scikit-learn (1.4.2)* [48].

**Fig 3:**
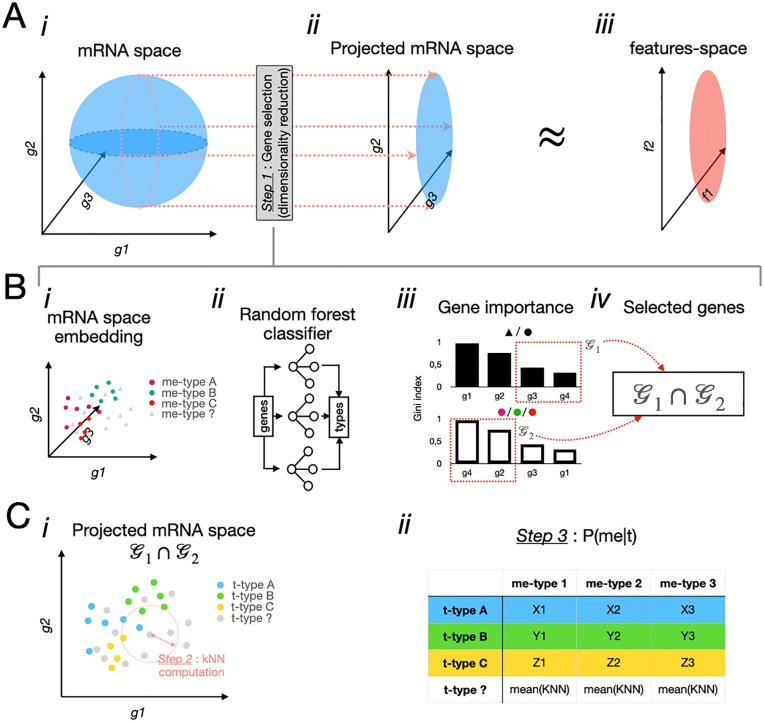
Stepwise construction of the extended probabilistic map. A. Principle overview***.*** (i) High-dimensional mRNA expression space is defined by many genes ***(g1, g2, g3)***. (ii) Gene selection and dimensionality reduction project the space into a subspace ***(g2, g3)*** that captures information relevant for predicting morphological and electrophysiological properties. (iii) This projected gene space approximates the functional feature space ***(f1, f2)*** used for label prediction. B. Detailing the gene selection step (***Step1***) using supervised models***.*** (i) Example embedding of neurons in mRNA space colored by me-type labels. (ii) Candidate genes are evaluated using Random Forest classifiers and regression models trained to predict me-labels. (iii) Feature importance (e.g., Gini importance) is calculated for each gene. (iv) The final set of selected genes is defined by the intersection of best predictors for me-types and genes with low region-predictive power. C. kNN-based mapping of uncovered t-types. (i) Neurons of uncovered t-types are projected into the selected gene subspace (G1∩G2). Distances between cells are computed using Euclidean metrics, and k-nearest neighbors (k = 10) are identified (***Step 2***). (ii) Probabilities for uncovered t-types are inferred by averaging the probabilities of their nearest neighbors, weighted by distance, yielding the extended probabilistic map P(me−type∣t−type) (***Step 3***).

Top-ranking genes most predictive of me-labels were identified based on importance scores derived from each model (e.g., Gini importance [50,51])(Fig 3B). A secondary model was then trained to predict the region of origin, and the 100 genes with the lowest scores in this context were identified. The intersection of these two gene sets formed a subspace optimized for predicting me-types while minimizing regional variability. The selected genes served as input to a k-nearest neighbors model (Fig 3Ci), where gene expression profiles were scaled, and Euclidean distance matrices were computed to identify the closest matching neuronal types. Probabilistic mappings for uncovered t-types were inferred by calculating the average of the nearest neighbors’ probabilities, weighted by the distance metrics (Fig 3Cii).

Model performance was assessed using 10-fold cross-validation (KFold for regression tasks, StratifiedKFold where label stratification was required) and two complementary error metrics: mean squared error (MSE) and mean absolute error (MAE). For each combination of algorithm and hyperparameters we recorded the average cross-validation MSE and MAE across folds and selected the combination that minimized these cross-validation metrics. This process also optimized the number of genes ($N_{genes}$) used to define the reduced space, as well as the number of neighbors ($N_{neighbors}$) required for calculating the probabilities of observing me-types for the uncovered t-types. This optimization step yielded the best results using the random forest algorithm with $N_{genes}=\text{1}\text{0}\text{0}$ and $N_{neighbors}$ = 10 (MSE = 0.08 and MAE = 0.027, S3 Fig).

This extended probabilistic map, integrating the newly extrapolated t-types, was used to later compute the me-type densities across the brain using $Densities(me-type_{i})=\sum_{j}P(me-type_{i}|t-type_{j})\;*\;Densities(t-type_{j})$.

#### Calculation of mRNA densities.

To assess the fidelity of this reduced representation, we next evaluated how the transformation from t-types to me-types affected regional mRNA density estimates. For verification (see Results, Fig 5B,5C), we computed mRNA densities based on either t-type or me-type densities. Specifically, mRNA densities from t-types were calculated as follows:


$$mRNA_{t}=Densities_{t}\cdot expression_{t},$$

where $expression_{t}$is a matrix of mRNA counts (with t-types as rows and genes as columns), $Densities_{t}$ is a matrix of t-type densities (with brain regions as rows and t-types as columns), and $mRNA_{t}$ is a matrix of mRNA densities (with brain regions as rows and genes as columns).

Similarly, mRNA densities can be derived from me-type densities as


$$mRNA_{me}=Densities_{me}\cdot expression_{me}$$

which can be expanded to


$$mRNA_{me}=P(me-type|t-type)\cdot Densities_{t}\cdot P(me-type|t-type)\cdot expression_{t}\times correction$$

where P(me−type∣t−type) represents the previously computed extended probabilistic map, and correction is an adjustment factor. This correction addresses the increase in density values that arises when converting 4,804 neuronal t-types into 458 functional me-types, which would otherwise lead to an overestimation of mRNA densities in me-types. The correction factor is defined as:


$$\begin{array}{c} orrection={\left(\frac{N_{me-types}}{N_{t-types}}\right)}^{\text{2}}={\left(\frac{\text{4}\text{5}\text{8}}{\text{4}\text{8}\text{0}\text{4}}\right)}^{\text{2}}\approx \text{0}.\text{0}\text{0}\text{9}\text{.} \end{array}$$

The square term results from the probabilistic map being applied twice in this calculation.

The error between both estimates was computed as $error=\frac{\left|mRNA_{t}-mRNA_{me}\right|}{mRNA_{t}}$.

#### Elbow method to determine the number of clusters.

To analyze the diversity of cell type compositions across brain regions (categorized by either t-types or me-types), we performed K-means clustering on UMAP-projected cell type density data (Fig 6Ai,6ii). The optimal number of clusters was determined using the Elbow Method, a heuristic that balances cluster compactness with model complexity [52,53]. This approach involves calculating the within-cluster sum of squares (WCSS) over a range of cluster counts, from 1 up to a predetermined maximum (in this case, 10 clusters). WCSS, which measures the total variance within each cluster, serves as an indicator of clustering compactness as clusters are added. When WCSS values are plotted against the number of clusters, the optimal cluster count is identified at the “elbow point”, where additional clusters yield diminishing reductions in WCSS. This elbow point represents a balance between minimizing intra-cluster variance and avoiding unnecessary model complexity.

**Fig 4:**
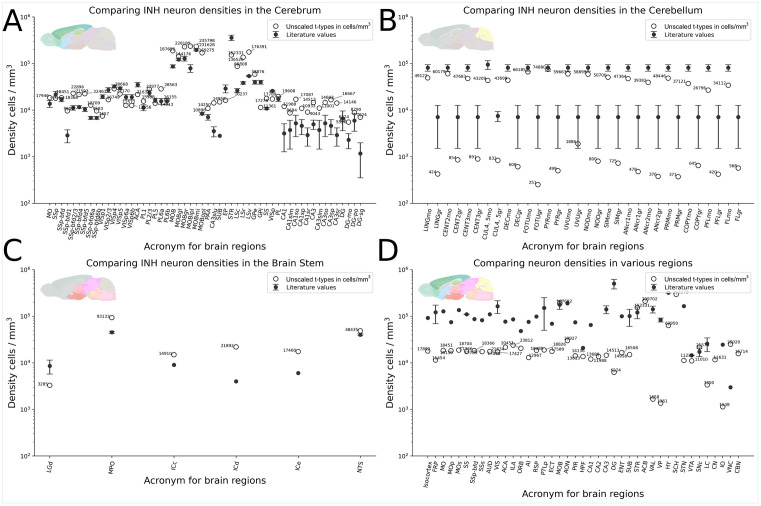
Comparison of predicted density with literature values. **A.** We extracted inhibitory neuron densities from the cerebrum from the unscaled atlas and compared them with inhibitory neuron numbers from the literature [7,38]. **B.** Inhibitory densities extracted from the cerebellum, and **C.** the brain stem. **D.** Comparison of total neuron density values from the scaled atlas with values from the literature [7]. Insets in the top-left corners show sampled areas, colored according to the annotation scheme used in the AIBS reference atlas. For regions with multiple reported literature values, we depict the variability using error bars. Acronyms are listed in S2 Table.

**Fig 5:**
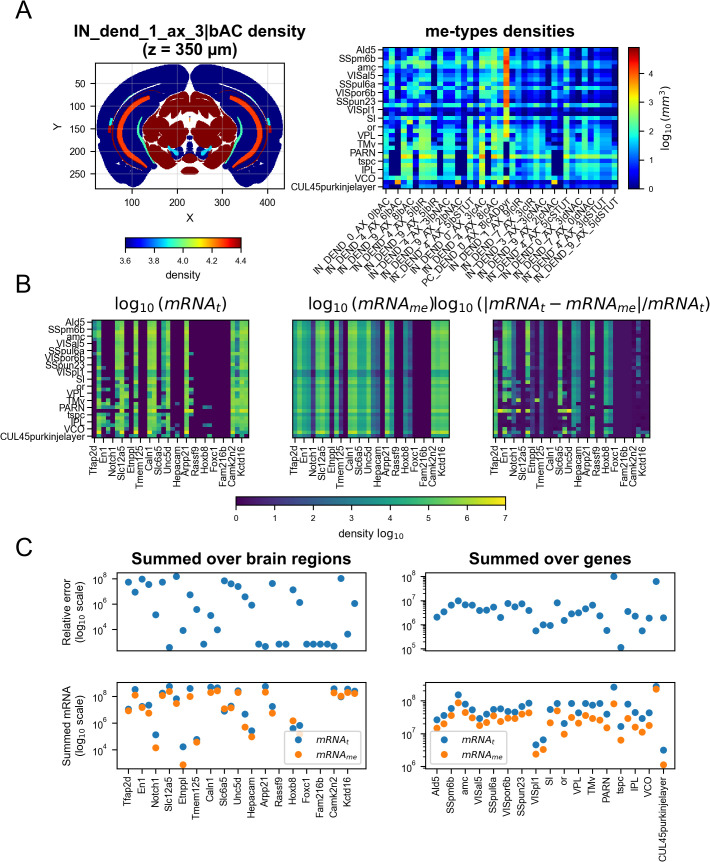
Probabilistic mapping application on t-type densities and validation of mRNA counts globally. **A.** Example of a coronal section of the density of the IN_dend_1_ax_3|bAC me-type, which preferentially maps to basket cells related t-types after applying the probabilistic mapping (*left*) and a heatmap (*right*) of obtained densities for a selection of brain regions (rows) and a selection of me-type (columns). For clarity, only a subset of me-types and brain regions are shown (including regions of CA1, DG, VIS, SS). **B.** mRNA counts obtained by multiplying cell types gene expression profiles with their densities (see Methods) in a selection brain regions and a selection of genes for t-types (*left*) and me-types (*middle*) and their relative error (*right*). **C.** Semilogarithmic plots of mRNA counts summed over regions (*left*) or genes (*right*). The top row shows the relative error, defined as $|mRNA_{t}\;-\;mRNA_{me}|\;/\;mRNA_{t}\;$ (log₁₀ scale), while the bottom row shows the corresponding summed mRNA counts (log₁₀ scale). Blue points correspond to values derived from transcriptomic t-type data ($mRNA_{t}$), and orange points to values obtained from morphological-electrophysiological me-type estimates ($mRNA_{me}$).

**Fig 6:**
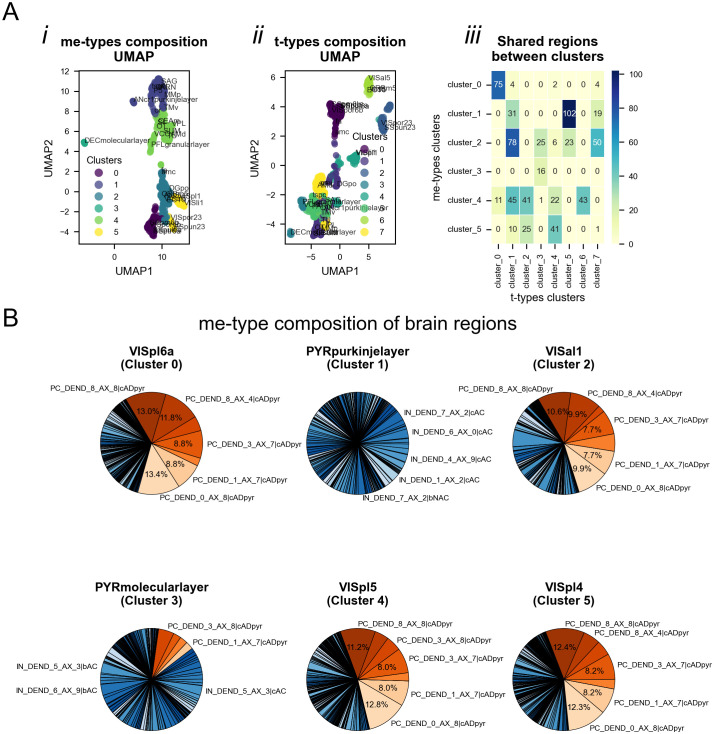
Brain region cell types composition. **A.** UMAP projection of brain regions based on (*i*) me-type and (*ii*) t-type density profiles. Each point represents one brain region, positioned according to its high-dimensional composition projected into a two-dimensional UMAP space (*UMAP1* and *UMAP2* are arbitrary axes that preserve relative similarity, not direct biological variables). Colors indicate k-means cluster assignments, which group regions with similar cellular compositions. (*iii*) Overlap matrix showing the percentage of brain regions shared between me-type based clusters (rows) and t-type based clusters (columns), with color intensity reflecting overlap magnitude. **B.** Exemplar region profiles: pie charts show the me-type composition of me-types in six representative brain regions chosen from distinct clusters. Only me-types comprising more than 5% of the brain region are displayed for clarity. Excitatory me-types are shown in shades of red, and inhibitory me-types in shades of blue. A complete color legend for all me-types is provided in the Supplementary Information.

#### Example use case: analyzing m- e-, and me-type densities.

To facilitate quantitative analysis of cell-type distributions and downstream modeling across the brain, we generated a comprehensive database encompassing all anatomical areas for which MERFISH data were available. For each region, we computed and stored cell densities (cells/mm³). These values were then organized into a brain-wide matrix, with cell types as rows and brain regions as columns, enabling efficient querying, direct comparison across regions, and statistical analyses (S3 Table, S4 Table). The same workflow was subsequently applied to morphological (m-type), electrophysiological (e-type), and joint morpho-electrical (me-type) classifications, thereby ensuring methodological consistency across modalities (S5 Table). All intermediate calculations and analysis code are publicly available in the GitHub repository (https://github.com/YannRoussel/probabilistic_mapping_extension).

It is important to note that most of these density estimates represent model-based predictions rather than independently validated measurements. Qualitative agreement with expected distributions is possible for some cell types, though. For example, the predicted distribution of cADpyr neurons is consistent with established anatomical knowledge, correctly localizing these cells to the cortex and hippocampus [11]. However, the limited availability of external quantitative datasets currently restricts broader validation across all cell types and regions. Our objective here was to provide quantitative estimates of cell-type densities for every brain region, while acknowledging the limitation that many of these values cannot yet be independently validated against existing literature.

To improve usability and anatomical interpretability, for every single m-, e, and me-types, we generated cross-sectional visualizations of the reconstructed three-dimensional brain within the Blue Brain Project’s enhanced CCFv3 annotation volume and reference framework (S8–S10 Figs). These allow users to spatially contextualize regional density estimates within a standardized coordinate system. Furthermore, we developed an accompanying tutorial notebook that guides users through the complete analysis pipeline: extracting regional densities from the database, reconstructing both two-dimensional slices and three-dimensional brain volumes, visualizing them through axial, coronal, and sagittal cross-sections, and integrating their own experimental datasets. Within this workflow, researchers can align their datasets to the canonical atlas, visualize it next to any number of cell types at any brain coordinates, and perform quantitative (density) comparisons to validate their findings, particularly in regions where prior literature is sparse or unavailable.

We also provide example code demonstrating how to query and extract data, group cell types into inhibitory and excitatory classes, and visualize t-, m-, e-, and me-type densities across defined anatomical subdivisions, e.g., to examine cumulative densities within laminar structures of the cortex or hippocampus. It should be noted that the current regionalization of the olfactory areas does not yet permit analysis of its laminar organization. Collectively, these resources transform the atlas from a static reference into a practical and extensible analytical framework for multimodal cell-type analysis.

## Results

### Data quality, technical considerations, and validation of density estimates

The densities presented in this atlas are derived from the MERFISH spatial transcriptomics dataset integrated into the Allen Brain Cell (ABC) Atlas, aligned to the extended and improved CCFv3 reference framework. While this dataset provides high-resolution transcriptomic cell type identification across 53 coronal slices and covers 670 of the 677 leaf regions in the mouse brain ontology, several technical factors influence the accuracy of the raw density estimates.

MERFISH-based cell detection consistently underestimates total cell numbers in highly dense brain regions, most notably the granular layers of the cerebellum (yielding only ~6.2 million cells and ~3.5 million neurons across the entire cerebellum) and the densely packed pyramidal and granule cell layers of the hippocampal formation (dentate gyrus, CA fields). These underestimations are attributable to well-documented limitations of in situ transcriptomics and image segmentation in crowded cellular environments, including optical crowding, probe saturation, limited gene detection per cell, and difficulties in resolving individual cell boundaries in small or tightly packed neurons.

Conversely, several regions exhibit higher-than-expected cell counts compared to classical isotropic fractionation literature values. Low-density areas (e.g., certain fiber tracts, cerebral nuclei, and olfactory regions) show consistent overestimation, with the olfactory areas reaching ~7 million neurons versus the expected 1–2 million. Possible contributing factors include segmentation artifacts (counting cell fragments or noise as cells), minor registration offsets that expand regional boundaries, inclusion of cells from adjacent areas, and occasional misclassification of non-neuronal nuclei as neurons due to marker thresholds.

White matter regions represent a special case. Raw MERFISH counts indicate that fiber tracts contain 61% of the cell type diversity observed in the entire cerebral cortex, despite neurons comprising only 8.81% of cells (with 91.18% non-neuronal). Quality control metrics provided by the ABC Atlas showed no significant differences between white and gray matter regions or between neuronal and non-neuronal types within white matter, and cell positions did not cluster abnormally near gray-white boundaries, suggesting that these observations are not primarily artifacts of gross misalignment. Nevertheless, the biological presence of neuronal somata in white matter remains debated in the literature, and these estimates should be interpreted cautiously pending further high-resolution validation.

On average, brain regions contained 4,819 cells (median 1,430), corresponding to 124 cell types per region, with leaf regions intersected by an average of 7.13 slices (median 5). Unscaled MERFISH estimates yielded a whole-brain total of ~92 million cells, substantially lower than the consensus literature value of 108.69 ± 16.25 million total cells [5].

To address these systematic discrepancies while preserving the relative cell type ratios and spatial patterns captured by MERFISH, we applied multiple scaling and correction strategies (detailed in Methods). Global scaling aligned total neuron counts to literature-derived targets (12.6 million in cerebral cortex, 41.8 million in cerebellum, 16.4 million elsewhere), density transplantation incorporated region-specific literature values where available (51 regions including striatum), and voxel-wise Nissl intensity corrections introduced intra-regional granularity using an average Nissl template derived from 734 postnatal day 56 C57BL/6J brains. Comparison of the two Nissl scaling variants (maximum-based vs. minimum-based) showed that the maximum-based approach produced higher densities across most regions, with the largest differences in cerebellum and select cortical areas, yet overall unscaled and scaled values exhibited very strong correlation.

After scaling, regional neuron and total cell counts showed markedly improved agreement with independent literature measurements (Fig 4 and Table 1), particularly in major brain divisions. These adjustments enable more accurate absolute quantification while retaining the high-resolution, method-consistent relative distributions provided by the MERFISH dataset. All scaling choices are modular and user-selectable in the accompanying computational pipeline.

**Table 1:** Total cell and neuron counts across the major brain regions, as estimated from MERFISH data. We aggregated the density values and transcriptomic cell type counts across major brain areas, expressed as the number of cells/mm³. See supplementary materials for the detailed table.

Brain area	Nr of cell types	Total sum of cells	Nr of neuron types	Sum of neurons
**Whole brain**	5272	92,231,205	5155	52,040,591
**Cerebral cortex**	3095	43,858,738	2989	29,125,071
**Isocortex**	946	28,535,494	870	19,101,627
**Cerebellum**	861	6,224,671	781	3,543,172
**Fiber tracts**	3174	10,371,674	3060	2,490,673
**Hippocampal formation**	1743	6,949,049	1653	3,890,269
**Hippocampus**	590	4,086,949	508	2,324,177
**Thalamus**	1682	3,260,859	1594	1,829,761
**Striatum**	1379	9,844,474	1297	7,114,754
**Olfactory areas**	1833	10,966,013	1738	7,099,982
**Cortical subplate**	761	1,440,177	697	920,486
**Cerebral nuclei**	1642	11,816,613	1557	8,020,503
**Interbrain**	2949	6,436,213	2846	3,552,739
**Midbrain**	2399	6,651,056	2317	2,870,715

### Regional transcriptomic cell densities and comparison with literature

After scaling, total neuron estimates across major brain divisions aligned closely with values derived from isotropic fractionation and other classical counting methods (Fig 4D). The cerebral cortex showed approximately 2.5-fold more neurons than predicted by primate scaling rules [5], while the isocortex exhibited a 1.5-fold increase. Thalamic and striatal neuron counts fell within or slightly above previously reported ranges, whereas hippocampal formation totals were consistent with existing literature despite under-sampling in dense layers.

Notably, white matter regions contained a surprisingly diverse set of cell types, representing 61% of the diversity observed across the entire cerebral cortex. These observations, combined with the improved agreement in scaled regional counts (Fig 4 and Table 1), highlight the atlas’s ability to capture both broad quantitative agreement with prior work and previously under-appreciated regional cellular diversity.

### Probabilistic mapping and validation

The probabilistic mapping approach successfully linked transcriptomic types (t-types) to morphological-electrophysiological types (me-types) using patch-seq datasets. Alignment of patch-seq cells to the Yao et al. reference taxonomy preserved major functional distinctions, with excitatory neurons mapping predominantly to cortical excitatory t-types and inhibitory neurons retaining their canonical family classifications (Pvalb, Vip, Sst) (S1 Fig).

Validation of the mapping pipeline showed satisfactory functional consistency across modalities. Conditional probabilities of e-types given t-types and morphological space coverage by patch-seq data were robust (S2 Fig). Extrapolation to t-types not directly sampled in patch-seq datasets was optimized using a random forest–based gene selection strategy (100 genes, k = 10 nearest neighbors), achieving low cross-validation error (MSE = 0.08, MAE = 0.027) (S3 Fig).

### Me-type densities and gene expression fidelity

Application of the probabilistic map to the scaled t-type density atlas produced whole-brain distributions for 458 me-types (Fig 5A). Comparison of predicted mRNA counts derived from t-type versus me-type densities revealed that me-type-based estimates were more uniform across brain regions, as expected from the reduction in cell type granularity (from 4,804 t-types to 458 me-types) (Fig 5B,5C). Relative errors remained generally consistent across regions and genes, with cumulative errors higher when summed over genes than over brain regions. These results indicate that the mapping preserves broad expression trends while introducing modest homogenization.

### Brain region cell type compositions

Clustering of brain regions based on me-type composition yielded six distinct groups, compared with eight groups when using the finer t-type composition (Fig 6Ai–6ii). The reduced number of clusters reflects some loss of granularity but still captured biologically meaningful organizational patterns. Overlap analysis showed bidirectional mapping between me-type and t-type clusters, with several t-type clusters corresponding to multiple me-type clusters and vice versa (Fig 6Aiii).

Representative brain regions from different clusters illustrated marked variation in me-type dominance: some areas were dominated by a few excitatory types (e.g., layer 6b of primary somatosensory cortex), while others showed greater diversity (e.g., ventral cochlear nucleus) (Fig 6B). This variation may be explained by the structural organization of cortical regions, which are layer-specific (e.g., layer 6b of the somatosensory primary cortex) and are populated by distinct excitatory me-types (e.g., PC_dend_1_ax_7|CADpyr, which is primarily found in cortical layers 5/6) [11,29,30]. These patterns suggest that the me-type framework effectively summarizes functional cell type organization across the brain despite the reduction in resolution.

## Discussion

This study presents a 3D atlas of estimated transcriptomic, morphological and electrophysiological cell type densities offering a multimodal perspective on diversity in the mouse brain. We used the complete mouse brain taxonomy and the MERFISH dataset from the ABC Atlas, an extended and improved CCFv3 annotation volume, and an array of corrective and scaling methods to first create a transcriptomic type (t-type) densities atlas. We then leveraged a probabilistic mapping approach to infer morpho-electrical (me-) types from t-types across brain regions.

### Enhancing interoperability between anatomical annotation systems

One of the key outcomes of this work is the projection of cell densities into a 3D reference space. The ABC Atlas introduced a hierarchical annotation system for their 3D reference space (CCFv3), where the tiling of parcellations reflects the resolution at which cells were identified, following the point-to-point mapping between the original MERFISH coordinate space and the CCF. This approach enabled users to aggregate cells based on parcellation assignments at varying anatomical levels. We reconciled differences between this new system (substructures, structures, divisions, categories, organs) and older anatomical annotations (leaf regions, intermediate and higher levels of the hierarchy, called parent regions).

However, none of these anatomical label reference systems are flawless. Some regions are either absent from the set of annotations or exist as annotations without assigned voxels. Furthermore, certain voxels could not be mapped to the lowest level of the hierarchy, leading to inconsistencies when calculating volumes. To address these limitations and ensure optimal interoperability between systems, we utilized the Blue Brain Project’s enhanced CCFv3 annotation volume and reference system (S4 Fig). By integrating additional metadata for each cell, users can navigate across different annotation systems. However, this approach comes with biological limitations, particularly in homogenizing regions where positional data was only available at higher levels of the hierarchy during the MERFISH registration process.

Overall, as datasets become increasingly large and complex, users from diverse scientific backgrounds need enhanced interoperability and compatibility.

### Addressing limitations in MERFISH cell density estimations: scaling techniques

One key finding of this work is that MERFISH slices struggle to accurately capture cellular densities in dense brain regions. Specifically, in areas such as the granular layers of the cerebellum, the olfactory bulb, and the hippocampus, we observed empty spaces between identified cells, where one would expect higher cell concentrations. This limitation is consistent across all slices. To address this, we introduced a series of scaling techniques to better model total cell numbers across the brain, in some cases increasing the estimated cell counts by up to twenty-fold. Similar to the annotation system, we took a balanced approach by offering multiple scaling methods to adjust the original cell counts. We compared unscaled and scaled cell numbers with available literature values [7] and developed a strategy for transplanting densities that accommodates a wide range of applications, research communities, and computational modeling efforts. To address poor data quality in densely packed brain regions, higher image resolution or increased magnification is recommended to enhance image quality and improve detection. Given the substantial influence of these brain regions on overall cell counts, increased data sampling may be warranted to ensure both functional relevance and accurate quantification of rare cell types.

Another important finding is that MERFISH-derived transcriptomic densities yield a different total cell count in the mouse brain compared to the neuron scaling rules for primate brains [5]. While certain cell-dense regions were found to have fewer cells than expected due to technical limitations, the majority of the brain exhibited significantly higher cell counts. These results support more recent results like the Blue Brain Project's Mouse Cell Atlas [7,38] and from the ABC Atlas [54–56]. These atlases have utilized advanced imaging and molecular labeling techniques to generate more granular data, revealing greater regional variability in cell densities. For instance, the Blue Brain Project’s Cell Atlas [38], with its whole-brain dataset and refined alignment algorithms, suggests higher localized neuron densities, especially in specific cortical layers and subcortical regions. Similarly, the Brain Initiative Cell Census atlases and other Nissl-stained slice datasets show local variations in cell densities that deviate from the scaling rule’s averages, highlighting how methodological advancements are uncovering more complexity in brain composition than previous scaling models suggested. High-resolution scRNA-seq and advanced stereological counting techniques have further revealed more intricate distributions of both neuronal and non-neuronal cell types, demonstrating higher cell densities, particularly in regions with specialized functions [55, 57].

### Re-evaluating neuronal populations in white matter from MERFISH and high-resolution imaging

One of the important findings from the generated neuron densities was the significant presence of cell bodies in white matter regions. Upon examining the QC values from [8], we found no significant differences between white and gray matter areas, nor between neuronal and non-neuronal cell types in the white matter. We assessed the positions of cell bodies and found no significant clustering near the boundaries between white and gray matter, suggesting no misalignment in the registration process to the reference atlas. In contrast, in brain regions adjacent to large white matter areas, which are more prone to signal spillover effects, the correct ratio of excitatory and inhibitory neurons, along with non-neuronal cells, was observed.

Studies have also highlighted the presence of notable neuronal populations in the brain white matter, challenging previous assumptions. For example, research has identified specific densities of NeuN-positive neurons in white matter regions, such as the cingulum and external capsule, with some studies revealing even higher densities in genetically modified models relevant to neurological conditions [58,59].

A high-resolution electron microscopy study has identified features of neuronal activity and axonal density, contributing to the growing understanding of white matter's role beyond just being a conduit for neural communication [60]. These newer approaches to cell counting, imaging, and genetic analysis indicate that neuron numbers in specific white matter tracts might indeed be higher than previously expected. This has prompted a closer re-evaluation of neuronal counts in mouse brain white matter.

Notably, high QC values from the ABC Atlas do not preclude noise arising from misalignment at the white/gray matter transition zones. Thus, the reported neuron densities should be interpreted with caution. Importantly, our aim was not to overwrite neuron values based on the presence or absence of specific cell types reported in other scientific publications, as done in some previous studies.

### Probabilistic mapping and cross-modal validation

A key assumption in our construction of the probabilistic map P(me−type|t−type) is the conditional independence of e-types and m-types given a t-type. This allowed us to approximate the joint distribution as the product P(m−type|t−type)×P(e−type|t−type). While convenient, this assumption may not fully capture potential dependencies between e-type and m-type labels. In reality, some e-types preferentially co-occur with specific m-types (e.g., fast-spiking behaviour of basket cells) beyond what is explained by their mutual dependence on t-types. However, due to the nature of the available data, this approximation was necessary: P(m−type|t−type) was obtained from direct predictions on neurons with available m-type labels, whereas P(e−type|t−type) was derived from a separate probabilistic mapping approach [47], since direct cell-level prediction of e-types was not possible. The potential mismatch between these two sources introduces some uncertainty in the estimated P(me−type|t−type), particularly in cases where strong m-e dependencies exist independently of t-type. The product approximation will tend to under-estimate the probability of strongly co-occurring me-pairs and to over-estimate unlikely combinations. The magnitude of this effect depends on the strength of residual dependencies and the abundance of the affected t-types. Consequently, broad, high-level distinctions (for example excitatory versus inhibitory) are expected to be relatively robust, whereas fine-grained me-subtypes should be interpreted with greater caution. We did not perform a direct empirical validation of the independence assumption because the comprehensive joint m/e/t labelled datasets required for that test are not available for the entire reference MERFISH set used here. However, our initial validation steps suggest that the probabilistic mapping preserves broad distinctions among cell types across transcriptomic, electrophysiological, and morphological modalities (S1 Fig). The alignment between patch-seq and reference t-type labels demonstrated satisfactory functional consistency, especially for major classifications, such as excitatory versus inhibitory neurons. This consistency aligns with previously reported mappings [28] and suggests that the mapping approach maintains fundamental cell type characteristics. However, it should be noted that the validation of morphological alignment relied on qualitative assessments due to limited quantitative data. Because of the described limitation we present the product-based P(me−type|t−type) as a pragmatic first-order estimate and recommend that future work use datasets containing joint m/e/t annotations (or apply synthetic benchmarks) to quantify residual dependencies and, where necessary, build joint estimators that relax the independence assumption.

### Challenges in extrapolating me-types to uncovered t-types

The high-dimensional, sparse, and multimodal nature of spatial omics data, presents a significant challenge in the field. One of the central goals of our approach was to generalize me-type predictions to t-types not represented in the patch-seq data. By identifying predictive genes for m-types and e-types while minimizing regional variability, we generated a subspace that allowed for extrapolation to other brain regions (Fig 1 *bottom panel*, Fig 3B,3C). Our optimization of gene counts and neighbor parameters for kNN analysis provided a robust basis for this extrapolation. However, due to the inherent variability in transcriptomic profiles across brain regions, this generalization may oversimplify certain cellular complexities, limiting predictive accuracy. Although this approach holds potential for extending cellular characterizations, the probabilistic extrapolations require further validation. Additionally, we propose a method to customize the me-type composition for specific regions by incorporating special types defined by the user (see S1 Text: *Special Regions Case*). This approach leverages expert knowledge to mitigate the limitations of the current generalization framework and enhance the biological relevance of the resulting composition.

### Limitations of mRNA density estimations from me-types

Our comparison of mRNA densities derived from t-types and me-types highlights both the utility and limitations of the probabilistic approach. While mRNA densities inferred from me-types were relatively uniform across brain regions, the reduction from 4,804 t-types to 458 me-types introduced some homogeneity that obscures finer transcriptomic distinctions (Fig 5B,5C). This reduction led to a relative error, especially for gene-specific counts, though overall regional compositions were less affected. These findings suggest that the model may adequately capture general expression trends but lacks the specificity to accurately reflect individual gene expression patterns. Therefore, this approach may be better suited for broad overviews of cell type distributions rather than precise transcriptomic reconstructions.

### Regional homogeneity in cell type compositions: me-types vs. t-types

Brain regions were grouped into six clusters based on me-type composition and eight clusters based on t-type composition, revealing underlying organizational patterns even with the reduced resolution of the me-type framework (Fig 6). This comparison highlights the trade-offs in using a simplified cell type classification. While t-type compositions provided greater specificity for distinguishing between brain regions, the me-type-based clustering effectively summarized cell type profiles into broader, more generalizable groups. The smaller number of me-type clusters suggests some loss of detail, potentially overlooking finer distinctions between regions. Nevertheless, the relatively modest difference in cluster count implies that the me-type framework retains biological relevance despite its reduced complexity.

### Comparison with existing atlases

Recent efforts, such as the comprehensive transcriptomic and spatial atlas by the AIBS, have combined scRNA-seq with MERFISH to hierarchically map cell types across the mouse brain [8]. Similarly, the ABC Atlas provides a platform for visualizing multimodal single-cell data [63], while resources like BrainTACO [64] and computational frameworks such as FuseMap [65] integrate transcriptomic, spatial, and connectivity modalities. New spatial methods such as Stereo-seq V2 have expanded coverage and throughput [66]. Building on previous approaches that mapped me-types to inhibitory markers [48], our work extends these efforts by incorporating transcriptomic, morphological, and electrophysiological data at a whole-brain scale. This integration bridges historical definitions of cell types with transcriptomics-based taxonomies, enabling a more comprehensive understanding of neuronal diversity and function than atlases limited to one or two modalities. Converting transcriptomic clusters into functional morphological-electrophysiological classifications also allows quantitative comparison with older cell counts and supports density-based modeling. Despite these advances, field-wide efforts face challenges, including resolution mismatches across modalities, difficulties in harmonizing datasets from different platforms, and the risk of bias in inferred cross-modal alignments.

Extending these efforts, by integrating voxel-based volumetric information, we move beyond 2D density maps to obtain true 3D distributions and absolute cell counts for each transcriptomic type across the brain. This framework provides several biological insights by jointly combining aligned slice-derived densities, morphological and transcriptomic class definitions, and complete volumetric annotations. Together, these components yield the first quantitative atlas in which me-/met-type abundance and identity can be directly compared across regions and depths of the brain. As a result, we can resolve volumetric gradients and regional differences in me-/met-type abundance, rather than artifacts arising from slice sampling or regional boundaries. Furthermore, we can quantify co-occurrence and spatial correlations between metabolic, electrophysiological, and transcriptomic features, and robustly identify rare or regionally restricted me-/met-types throughout the anatomical volume. Finally, by expressing me- and met-type densities in units of cells per voxel, our approach enables direct, quantitative comparisons of energetic and electrophysiological demands across regions and cell classes, analyses that were previously infeasible with 2D projections or non-voxelized 3D maps.

### Accessibility of the atlas

Computational workflows in neuroscience often face challenges in reproducibility and integration [61,62]. All code and processed data generated in this project are publicly available to support transparency, reproducibility, and community-driven extensions. To make the data accessible to a broad range of users, we provide both command-line interfaces (CLI) and Jupyter Notebooks that cover the full computational workflow, from raw data processing to visualization and analysis. Additionally, a tutorial is included to guide new users through accessing and interacting with the dataset, ensuring that both computational biologists and experimentalists can readily explore and build upon our work.

The electrophysiological, morphological, and transcriptomic cell types in our work are organized into high-resolution 3D brain atlases that enable voxel-level comparisons of single-cell type expression across the entire brain. These atlases are precisely aligned with both the AIBS’s reference atlas and the Blue Brain Project’s updated brain atlas, which includes extended cerebellar, olfactory, and medullary regions. This alignment allows for direct regional and voxel-wise comparisons of met-type density and expression patterns. Furthermore, the data is designed for seamless visualization with widely used software tools such as ITK-Snap, Fiji, MITK, napari, NiftyNet/MONAI, and 3D Slicer, making it accessible and interpretable across a variety of analysis platforms.

### Applications of the atlas

The atlas's output can be directly used for large-scale brain simulations. For instance, earlier versions of this atlas have been applied to simulations of the somatosensory cortex [34,35]. These simulations are comprehensive, taking into account the brain's morphological and electrical properties as well as its neuron composition. This represents a promising direction for future research, potentially within the scope of the new Open Brain Institute (https://www.openbraininstitute.org).

An important yet unexplored avenue in our work is the analysis of me-type cell presence and density across specific brain regions. While our project’s main objective was to successfully generate quantitative data on these cell types for brain atlases, we did not pursue a detailed investigation of their spatial distribution or functional implications. Our atlas can serve as a reference for study of the presence or absence of met-types and their relative densities. Given the current lack of comprehensive data in this area, future work could leverage our dataset as a comparative baseline to uncover region-specific patterns and generate new insights into the organization of these cell types. Similarities in cell composition between different regions might suggest they have similar functions.

### Future directions

As the availability of multimodal electrophysiological, morphological, and transcriptomic data continues to expand, the adaptable pipeline developed in this study can be readily extended to incorporate these new datasets. The strength of this approach lies in its ability to integrate diverse data derived from various techniques, as all information is consistently mapped to cellular density. Furthermore, anticipated advancements in spatial transcriptomics technologies, such as Slide-seq [63] and High-Definition Spatial Transcriptomics [64], hold the potential to significantly enhance the resolution and accuracy of future atlases. Finally, the use of transcriptomic types allows linking of features measured separately in different laboratories to the cell type, and potentially even comparison across species. We believe that quantitative atlases of this nature are useful tools for generating novel hypotheses amenable to experimental validation, thereby guiding and accelerating future neuroscientific research.

## Conclusion

This study presents a probabilistic framework for creating a 3D atlas of t-, m-, and e-type densities. Our approach effectively integrates multimodal data and produces biologically relevant regional density estimates. The atlas and pipeline provide a flexible, scalable tool for exploring large-scale cell type distributions. By unifying transcriptomic, morphological, and electrophysiological data in a spatially resolved atlas, this work provides a foundation for multiscale modeling of brain function and disease.

## Supporting information

S1 Table Leaf regions not covered by MERFISH slices. (CSV)

S2 Table All acronyms for Fig 4 and S7 Fig. Acronyms and region names follow the nomenclature used in the Allen Institute for Brain Science (AIBS) reference atlas.
(CSV)

S3 Table Main cell type densities (T-types) summarized across the major brain regions. (XLSX)

S4 Table Main cell type densities (T-types) summarized across the major brain regions. (CSV)

S5 Table Mean cell type densities (M-, E-, Me-types) by leaf and non-leaf regions. (CSV)

S1 Text Supplementary information. (DOCX)

S1 Fig Validation of t-types alignment method. Probability maps highlighting the t-type alignment between native labels (given by the patch-seq datasets) with the assigned reference t-types [54]. Excitatory t-types are on the top row, inhibitory on the bottom. Probabilities of having a t-type from [8] given a native label are on the left, while probabilities of observing a native label given a t-type from [8] are shown on the right.
(TIFF)

S2 Fig Validation of electrophysiological and morphological mappings. **A.** Probabilities of observing a canonical e-type given a t-type from [8] in the patch-seq dataset from [30] (left). Probabilities of observing e-types as defined by [26] given canonical e-types (right, reproduced from [48]). bSTUT, cSTUT, dNAC and dSTUT mapped preferentially to fast spiking cells (FS) while all the other mapped mostly to non fast spiking neurons (nFS) **B.** Coverage of the [29] dataset (left) and the patch-seq dataset from [30] of the embedding of the canonical morphological space are defined by the reference morphological dataset (labeled as “BBP”).
(TIFF)

S3 FigOptimization of the pipeline parameters.($N_{genes}$, $N_{neighbors}$, Clustering algorithm). Mean squared error (MSE, top) and mean absolute error (MAE, bottom) as a function of $N_{neighbors}$ for $\begin{array}{c} N_{genes}=\text{50},\;\text{100},\;\text{500},\;\text{1000 and 10000} \end{array}$ and for multiple machine learning algorithms combination for e-features space and m-types space gene selection for the 10-fold cross-validation process. Several combinations were tried: lasso and random forest (**A**), random forest and random forest (**B**), lasso and mutual info (**C**), random forest and mutual info (**D**).(TIFF)

S4 Fig Different annotation atlas versions can be used to create various iterations of transcriptomic atlases. **A.** Sagittal (y = 200) and coronal (x = 315) sections of the CCFv3 annotation volume (from the AIBS (x = 300) and the **B.** extended CCFv3 annotation volume from the literature [33]. The legend annotation colors match those in the Allen Institute reference atlas. Resolution: 25 μm^3^ voxel size.
(TIFF)

S5 Fig 3D transcriptomic atlases generated with scaled densities. Sagittal (y = 200) and coronal (x = 300) sections of scaled density volumes including all cell types: **A.** Scaled to match total cell numbers reported in [5]; **B.** Scaled to match total cell numbers from [5] with transplant adjustment; **C.** Regional total cell type densities scaled to reflect differences in Nissl staining intensity. Resolution: 25 μm^3^ voxel size.
(TIFF)

S6 Fig Nissl scaling of 3D transcriptomic atlases. Sagittal (y = 200) and coronal (x = 300) sections of scaled density volumes (total cell): **A.** All regional total cell densities are scaled to reflect differences in Nissl intensity, scaled with the minimum Nissl intensity; **B.** All regional total cell densities are scaled to reflect differences in Nissl intensity, scaled with the maximum Nissl intensity equals 4 million cells/mm^3^; **C.** Nissl granularity was added to **A** to emulate variance within each region. Resolution: 25 μm^3^ voxel size.
(TIFF)

S7 Fig Comparison of estimated neuron densities with values from the literature. **A.** We extracted inhibitory neuron densities from the cerebrum from the scaled atlas and compared them with inhibitory neuron numbers from the literature [7,38]. **B.** Inhibitory densities extracted from the cerebellum, and **C.** the brain stem. Comparison of total neuron density values from the scaled atlas with values from the literature [7,38]. Insets in the top-left corners show sampled areas, colored according to the annotation scheme used in the AIBS reference atlas. For regions with multiple reported literature values, we depict the variability using error bars. Acronyms are listed in S2 Table.
(TIFF)

S8 Fig 3D atlases of e-types generated with scaled densities. Coronal (x = 300) and sagittal (y = 200) sections of scaled density volumes of all 11 e-types. Density values were projected in the extended and improved CCFv3 annotation volume. Resolution: 25 μm^3^ voxel size. Colorbars show the number of cells / mm^3^ (rounded) for every panel. Abbreviations: cADpyr: continuous adapting pyramidal neuron (excitatory), bAC: bursting accommodating (inhibitory), bIR: bursting irregular spiking (inhibitory), bNAC: bursting non-adapting (inhibitory), bSTUT: bursting stuttering (inhibitory), cAC: continuous adapting (inhibitory), cIR: continuous irregular spiking (inhibitory), cNAC: continuous non-adapting (inhibitory), cSTUT: continuous stuttering (inhibitory), dNAC: delayed non-accommodating (inhibitory), dSTUT: delayed stuttering neuron (inhibitory)
(TIFF)

S9 Fig 3D atlases of m-types generated with scaled densities. Coronal (x = 300) and sagittal (y = 200) sections of scaled density volumes of all m-types. Density values were projected in the extended and improved CCFv3 annotation volume. Resolution: 25 μm^3^ voxel size. Colorbars show the number of cells / mm^3^ (rounded) for every panel.
(TIFF)

S10 Fig 3D atlases of me-types generated with scaled densities. Coronal (x = 300) and sagittal (y = 200) sections of scaled density volumes of all me-types. Density values were projected in the extended and improved CCFv3 annotation volume. Resolution: 25 μm^3^ voxel size. Colorbars show the number of cells / mm^3^ (rounded) for every panel.
(TIFF)

## References

[1] Cook SJ, Jarrell TA, Brittin CA, Wang Y, Bloniarz AE, Yakovlev MA, et al. Whole-animal connectomes of both Caenorhabditis elegans sexes. Nature. 2019;571(7763):63–71. doi: 10.1038/s41586-019-1352-7 31270481 PMC6889226

[2] Ryan K, Lu Z, Meinertzhagen IA. The CNS connectome of a tadpole larva of Ciona intestinalis (L.) highlights sidedness in the brain of a chordate sibling. Elife. 2016;5:e16962. doi: 10.7554/eLife.16962 27921996 PMC5140270

[3] Verasztó C, Jasek S, Gühmann M, Bezares-Calderón LA, Williams EA, Shahidi R. Whole-body connectome of a segmented annelid larva. eLife. 2024. 10.7554/eLife.97964 PMC12387772 40862480

[4] Schlegel P, Yin Y, Bates AS, Dorkenwald S, Eichler K, Brooks P, et al. Whole-brain annotation and multi-connectome cell typing of Drosophila. Nature. 2024;634(8032):139–52. doi: 10.1038/s41586-024-07686-5 39358521 PMC11446831

[5] Herculano-Houzel S, Mota B, Lent R. Cellular scaling rules for rodent brains. Proc Natl Acad Sci U S A. 2006;103(32):12138–43. doi: 10.1073/pnas.0604911103 16880386 PMC1567708

[6] Petilla Interneuron Nomenclature Group, Ascoli GA, Alonso-Nanclares L, Anderson SA, Barrionuevo G, Benavides-Piccione R, et al. Petilla terminology: nomenclature of features of GABAergic interneurons of the cerebral cortex. Nat Rev Neurosci. 2008;9(7):557–68. doi: 10.1038/nrn2402 18568015 PMC2868386

[7] Erö C, Gewaltig M-O, Keller D, Markram H. A Cell Atlas for the Mouse Brain. Front Neuroinform. 2018;12:84. doi: 10.3389/fninf.2018.00084 30546301 PMC6280067

[8] Yao Z, van Velthoven CTJ, Kunst M, Zhang M, McMillen D, Lee C, et al. A high-resolution transcriptomic and spatial atlas of cell types in the whole mouse brain. Nature. 2023;624(7991):317–32. doi: 10.1038/s41586-023-06812-z 38092916 PMC10719114

[9] Zhang M, Pan X, Jung W, Halpern AR, Eichhorn SW, Lei Z, et al. Molecularly defined and spatially resolved cell atlas of the whole mouse brain. Nature. 2023;624(7991):343–54. doi: 10.1038/s41586-023-06808-9 38092912 PMC10719103

[10] Tasic B, Menon V, Nguyen TN, Kim TK, Jarsky T, Yao Z, et al. Adult mouse cortical cell taxonomy revealed by single cell transcriptomics. Nat Neurosci. 2016;19(2):335–46. doi: 10.1038/nn.4216 26727548 PMC4985242

[11] Tasic B, Yao Z, Graybuck LT, Smith KA, Nguyen TN, Bertagnolli D, et al. Shared and distinct transcriptomic cell types across neocortical areas. Nature. 2018;563(7729):72–8. doi: 10.1038/s41586-018-0654-5 30382198 PMC6456269

[12] Langlieb J, Sachdev NS, Balderrama KS, Nadaf NM, Raj M, Murray E, et al. The molecular cytoarchitecture of the adult mouse brain. Nature. 2023;624(7991):333–42. doi: 10.1038/s41586-023-06818-7 38092915 PMC10719111

[13] Zeng H. What is a cell type and how to define it?. Cell. 2022;185(15):2739–55. doi: 10.1016/j.cell.2022.06.031 35868277 PMC9342916

[14] Poulin J-F, Tasic B, Hjerling-Leffler J, Trimarchi JM, Awatramani R. Disentangling neural cell diversity using single-cell transcriptomics. Nat Neurosci. 2016;19(9):1131–41. doi: 10.1038/nn.4366 27571192

[15] Regev A, Teichmann SA, Lander ES, Amit I, Benoist C, Birney E, et al. The Human Cell Atlas. eLife. 2017;6:e27041. doi: 10.7554/eLife.27041 PMC5762154 29206104

[16] Shapiro E, Biezuner T, Linnarsson S. Single-cell sequencing-based technologies will revolutionize whole-organism science. Nat Rev Genet. 2013;14(9):618–30. doi: 10.1038/nrg3542 23897237

[17] Lacar B, Linker SB, Jaeger BN, Krishnaswami SR, Barron JJ, Kelder MJE, et al. Nuclear RNA-seq of single neurons reveals molecular signatures of activation. Nat Commun. 2016;7:11022. doi: 10.1038/ncomms11022 27090946 PMC4838832

[18] Habib N, Avraham-Davidi I, Basu A, Burks T, Shekhar K, Hofree M, et al. Massively parallel single-nucleus RNA-seq with DroNc-seq. Nat Methods. 2017;14(10):955–8. doi: 10.1038/nmeth.4407 28846088 PMC5623139

[19] Chen KH, Boettiger AN, Moffitt JR, Wang S, Zhuang X. RNA imaging. Spatially resolved, highly multiplexed RNA profiling in single cells. Science. 2015;348(6233):aaa6090. doi: 10.1126/science.aaa6090 25858977 PMC4662681

[20] Fernández-Moya SM, Ganesh AJ, Plass M. Neural cell diversity in the light of single-cell transcriptomics. Transcription. 2023;14(3–5):158–76. doi: 10.1080/21541264.2023.2295044 38229529 PMC10807474

[21] Heumos L, Schaar AC, Lance C, Litinetskaya A, Drost F, Zappia L, et al. Best practices for single-cell analysis across modalities. Nat Rev Genet. 2023;24(8):550–72. doi: 10.1038/s41576-023-00586-w 37002403 PMC10066026

[22] Yano Y, Chiba T, Asahara H. Analysis of the Mouse Y Chromosome by Single-Molecule Sequencing With Y Chromosome Enrichment. Front Genet. 2020;11:406. doi: 10.3389/fgene.2020.00406 32457799 PMC7221202

[23] Nanda AS, Wu K, Irkliyenko I, Woo B, Ostrowski MS, Clugston AS, et al. Direct transposition of native DNA for sensitive multimodal single-molecule sequencing. Nat Genet. 2024;56(6):1300–9. doi: 10.1038/s41588-024-01748-0 38724748 PMC11176058

[24] ABCA. https://portal.brain-map.org/atlases-and-data/bkp/abc-atlas. Accessed 2024.

[25] Lin S, Wang Z, Cui Y, Zou Q, Han C, Yan R. Bridging the Dimensional Gap from Planar Spatial Transcriptomics to 3D Cell Atlases. Cold Spring Harbor Laboratory. doi: 10.1101/2024.12.06.627127 2024. Accessed 2025 July 14. 41476112

[26] Han L, Liu Z, Jing Z, Liu Y, Peng Y, Chang H. Single-cell spatial transcriptomic atlas of the whole mouse brain. Neuron. 2025. 10.1016/j.neuron.2025.02.015 40132589

[27] Wang N, Maharjan S, Tsai AP, Lin PB, Qi Y, Wallace A, et al. Integrating multimodality magnetic resonance imaging to the Allen Mouse Brain Common Coordinate Framework. NMR Biomed. 2023;36(5):e4887. doi: 10.1002/nbm.4887 36454009 PMC10106385

[28] Gouwens NW, Sorensen SA, Berg J, Lee C, Jarsky T, Ting J, et al. Classification of electrophysiological and morphological neuron types in the mouse visual cortex. Nat Neurosci. 2019;22(7):1182–95. doi: 10.1038/s41593-019-0417-0 31209381 PMC8078853

[29] Gouwens NW, Sorensen SA, Baftizadeh F, Budzillo A, Lee BR, Jarsky T, et al. Integrated Morphoelectric and Transcriptomic Classification of Cortical GABAergic Cells. Cell. 2020;183(4):935–953.e19. doi: 10.1016/j.cell.2020.09.057 33186530 PMC7781065

[30] Scala F, Kobak D, Bernabucci M, Bernaerts Y, Cadwell CR, Castro JR, et al. Phenotypic variation of transcriptomic cell types in mouse motor cortex. Nature. 2021;598(7879):144–50. doi: 10.1038/s41586-020-2907-3 33184512 PMC8113357

[31] Ramaswamy S, Courcol J-D, Abdellah M, Adaszewski SR, Antille N, Arsever S, et al. The neocortical microcircuit collaboration portal: a resource for rat somatosensory cortex. Front Neural Circuits. 2015;9:44. doi: 10.3389/fncir.2015.00044 26500503 PMC4597797

[32] Markram H, Muller E, Ramaswamy S, Reimann MW, Abdellah M, Sanchez CA, et al. Reconstruction and simulation of neocortical microcircuitry. Cell. 2015;163:456–92. doi: 10.1016/j.cell.2015.09.029 26451489

[33] Teeters JL, Godfrey K, Young R, Dang C, Friedsam C, Wark B, et al. Neurodata Without Borders: Creating a Common Data Format for Neurophysiology. Neuron. 2015;88(4):629–34. doi: 10.1016/j.neuron.2015.10.025 26590340

[34] Reimann MW, Bolaños-Puchet S, Courcol J-D, Santander DE, Arnaudon A, Coste B, et al. Modeling and Simulation of Neocortical Micro- and Mesocircuitry. Part I: Anatomy. eLife. eLife Sciences Publications Limited; 2024;13. doi: 10.7554/eLife.99688.2 PMC12818870 41556767

[35] Isbister JB, Ecker A, Pokorny C, Bolaños-Puchet S, Santander DE, Arnaudon A. Modeling and simulation of neocortical micro- and mesocircuitry. Part II: physiology and experimentation. bioRxiv. 2024. doi: 10.1101/2023.05.17.541168

[36] Piluso S, Verasztó C, Carey H, Delattre É, L’Yvonnet T, Colnot É, et al. An extended and improved CCFv3 annotation and Nissl atlas of the entire mouse brain. Imaging Neurosci (Camb). 2025;3. doi: 10.1162/imag_a_00565 40800985 PMC12319842

[37] Modat M, Cash DM, Daga P, Winston GP, Duncan JS, Ourselin S. Global image registration using a symmetric block-matching approach. J Med Imaging (Bellingham). 2014;1(2):024003. doi: 10.1117/1.JMI.1.2.024003 26158035 PMC4478989

[38] Rodarie D, Verasztó C, Roussel Y, Reimann M, Keller D, Ramaswamy S, et al. A method to estimate the cellular composition of the mouse brain from heterogeneous datasets. PLoS Comput Biol. 2022;18(12):e1010739. doi: 10.1371/journal.pcbi.1010739 36542673 PMC9838873

[39] Wolf FA, Angerer P, Theis FJ. SCANPY: large-scale single-cell gene expression data analysis. Genome Biol. 2018;19(1):15. doi: 10.1186/s13059-017-1382-0 29409532 PMC5802054

[40] McInnes L, Healy J, Saul N, Großberger L. UMAP: Uniform Manifold Approximation and Projection. J Open Source Softw. 2018;3:861. doi: 10.21105/joss.00861

[41] Wu C, Macleod I, Su AI. BioGPS and MyGene.info: organizing online, gene-centric information. Nucleic Acids Res. 2013;41(Database issue):D561–5. doi: 10.1093/nar/gks1114 23175613 PMC3531157

[42] Berchet A, Arnaudon A, alex4200. BlueBrain/morphology-workflows: 0.12.1. Zenodo. 2024. doi: 10.5281/ZENODO.10809196

[43] Kanari L, Dłotko P, Scolamiero M, Levi R, Shillcock J, Hess K. A Topological Representation of Branching Neuronal Morphologies. Neuroinformatics. 2018;16:3–13. doi: 10.1007/s12021-017-9341-1 28975511 PMC5797226

[44] Kanari L, Schmidt S, Casalegno F, Delattre E, Banjac J, Negrello T, et al. Deep learning for classifying neuronal morphologies: combining topological data analysis and graph neural networks. Neuroscience. 2024.

[45] Adams H, Emerson T, Kirby M, Neville R, Peterson C, Shipman P. Persistence Images: A Stable Vector Representation of Persistent Homology. J Mach Learn Res. 2017;18:1–35.

[46] Kanari L, Ramaswamy S, Shi Y, Morand S, Meystre J, Perin R, et al. Objective Morphological Classification of Neocortical Pyramidal Cells. Cereb Cortex. 2019;29(4):1719–35. doi: 10.1093/cercor/bhy339 30715238 PMC6418396

[47] Roussel Y, Verasztó C, Rodarie D, Damart T, Reimann M, Ramaswamy S, et al. Mapping of morpho-electric features to molecular identity of cortical inhibitory neurons. PLoS Comput Biol. 2023;19(1):e1010058. doi: 10.1371/journal.pcbi.1010058 36602951 PMC9815626

[48] Pedregosa F, Varoquaux G, Gramfort A, Michel V, Thirion B, Grisel O. Scikit-learn: Machine Learning in Python. J Mach Learn Res. 2011;12:2825–30.

[49] Roussel Y, Verasztó C, Rodarie D, Damart T, Reimann M, Ramaswamy S. Mapping of morpho-electric features to molecular identity of cortical inhibitory neurons. Neuroscience. 2021. 10.1371/journal.pcbi.1010058 PMC9815626 36602951

[50] Breiman L. Random Forests. Mach Learn. 2001;45:5–32. doi: 10.1023/A:1010933404324

[51] Nembrini S, König IR, Wright MN. The revival of the Gini importance? Bioinformatics. 2018;34(21):3711–8. doi: 10.1093/bioinformatics/bty373 29757357 PMC6198850

[52] Thorndike RL. Who Belongs in the Family? Psychometrika. 1953;18(4):267–76. doi: 10.1007/bf02289263

[53] Ketchen Jr. DJ, Shook CL. The application of cluster analysis in strategic management research: an analysis and critique. Strat Mgmt J. 1996;17(6):441–58. doi: 10.1002/(sici)1097-0266(199606)17:6<441::aid-smj819>3.0.co;2-g

[54] Wang Q, Ding S-L, Li Y, Royall J, Feng D, Lesnar P, et al. The Allen Mouse Brain Common Coordinate Framework: A 3D Reference Atlas. Cell. 2020;181(4):936–953.e20. doi: 10.1016/j.cell.2020.04.007 32386544 PMC8152789

[55] Yao Z, Liu H, Xie F, Fischer S, Adkins RS, Aldridge AI, et al. A transcriptomic and epigenomic cell atlas of the mouse primary motor cortex. Nature. 2021;598(7879):103–10. doi: 10.1038/s41586-021-03500-8 34616066 PMC8494649

[56] Callaway EM, Dong HW, Ecker JR, Hawrylycz MJ, BRAIN Initiative Cell Census Network (BICCN). A multimodal cell census and atlas of the mammalian primary motor cortex. Nature. 2021;598:86–102. doi: 10.1038/s41586-021-03950-0 34616075 PMC8494634

[57] Shi H, He Y, Zhou Y, Huang J, Maher K, Wang B, et al. Spatial atlas of the mouse central nervous system at molecular resolution. Nature. 2023;622(7983):552–61. doi: 10.1038/s41586-023-06569-5 37758947 PMC10709140

[58] Suarez-Sola ML. Neurons in the white matter of the adult human neocortex. Front Neuroanat. 2009;3. 10.3389/neuro.05.007.2009 PMC2697018 19543540

[59] Tsai S-H, Tsao C-Y, Lee L-J. Altered White Matter and Layer VIb Neurons in Heterozygous Disc1 Mutant, a Mouse Model of Schizophrenia. Front Neuroanat. 2020;14:605029. doi: 10.3389/fnana.2020.605029 33384588 PMC7769951

[60] Korogod N, Petersen CCH, Knott GW. Ultrastructural analysis of adult mouse neocortex comparing aldehyde perfusion with cryo fixation. Elife. 2015;4:e05793. doi: 10.7554/eLife.05793 26259873 PMC4530226

[61] Martone ME. The past, present and future of neuroscience data sharing: a perspective on the state of practices and infrastructure for FAIR. Front Neuroinform. 2024;17:1276407. doi: 10.3389/fninf.2023.1276407 38250019 PMC10796549

[62] Manninen T, Aćimović J, Havela R, Teppola H, Linne M-L. Challenges in Reproducibility, Replicability, and Comparability of Computational Models and Tools for Neuronal and Glial Networks, Cells, and Subcellular Structures. Front Neuroinform. 2018;12:20. doi: 10.3389/fninf.2018.00020 29765315 PMC5938413

[63] Stickels RR, Murray E, Kumar P, Li J, Marshall JL, Di Bella DJ, et al. Highly sensitive spatial transcriptomics at near-cellular resolution with Slide-seqV2. Nat Biotechnol. 2021;39(3):313–9. doi: 10.1038/s41587-020-0739-1 33288904 PMC8606189

[64] Vickovic S, Eraslan G, Salmén F, Klughammer J, Stenbeck L, Schapiro D, et al. High-definition spatial transcriptomics for in situ tissue profiling. Nat Methods. 2019;16(10):987–90. doi: 10.1038/s41592-019-0548-y 31501547 PMC6765407

[65] He Y, Sheng H, Shi H, Wang WX, Tang Z, Liu J, et al. Towards a universal spatial molecular atlas of the mouse brain. bioRxiv; 2024. p. 2024.05.27.594872. doi: 10.1101/2024.05.27.594872

[66] Chen A, Liao S, Cheng M, Ma K, Wu L, Lai Y, et al. Spatiotemporal transcriptomic atlas of mouse organogenesis using DNA nanoball-patterned arrays. Cell. Elsevier; 2022;185:1777–1792.e21. doi: 10.1016/j.cell.2022.04.003 35512705

