# Supplementary material for: A multimodal spatial atlas of transcriptomic, morphological, and electrophysiological cell type densities in the mouse brain
Source: PLoS Comput Biol. 2026 Mar 24;22(3):e1014106. doi: 10.1371/journal.pcbi.1014106 (PMC13120702; doi:10.1371/journal.pcbi.1014106)

# Supplementary information

##

## Supplementary Methods

**File format**

To store cell type densities, one can easily access every parcellation for the presence (or absence) of its cell types. It is also trivial to look at each cell type and list all parcellations where they are present. The difference compared to [[8]](https://www.zotero.org/google-docs/?NlKVm3) is the estimated density values in cells / mm^3^. For ease of use we generated 3D atlases for every cell type on the cluster level using the extended CCFv3 annotation volume. For any given region the voxels inherit the average cell density of the region. The implicit knowledge of the cell type atlas is that the absence of a cell type is similarly strong information. Thus to make calculations simple, we set voxels outside of the brain to np.nan, marking a clear difference between not present and not possible. The resulting 3D arrays are stored as *nrrd* files.

For in-between calculations, e.g. for cell type / region information which are stored in a dict of dataframes we stored the information in pickle format.

Total cell counts were calculated for every region by multiplying the density values with the region's total voxel count. This information is useful for comparing them with literature values where total cell counts were estimated. Storing this information in a single pickle file is the most convenient.

Finally, once density information is available in every leaf region, larger regions (higher in the anatomical hierarchy) can be readily calculated by summing up leaf regions which are part of a larger brain structure. This way, we can estimate densities in all levels of the anatomical hierarchy for all cell types, and larger cell groups, e.g. inhibitory, excitatory neurons or non-neuronal cells and store it as a table in a csv file.

**CCFv3 - Annotation volumes**

The mouse brain reference atlas or CCFv3 annotation volume, derived from 1,675 mouse brains, provides the ideal template for a 3D mouse brain atlas. Its smooth surface and expert validation make it one of the best and most up-to-date resources in the field. However, to ensure flexibility and future developments, we designed the pipeline in such a way that any type of cell atlas can be used as a template to generate a 3D atlas populated with comprehensive density data. To create a 3D brain representation of every cell type we used the extended version of the CCFv3 annotation volume (Supplementary Fig. 4). This comes with the added benefit of providing coverage of the entire brain. This atlas has more complete versions of both the olfactory bulb, the cerebellum, and the medulla, while it still adheres to the hierarchical structure of the common coordinate framework version 3 from the AIBS. As the rostral dimensions are higher, the 0 origin coordinate is shifted by 350 μm compared to the AIBS CCFv3 annotation volume.

Average cell densities were projected into the reference atlas space, and each voxel inherited a density value for any given cell type, based on the average value of all data from the brain slices covering the region the voxel belonged to. We used the 25 μm^3^ voxel size version to speed up calculations. However, as the pipeline calculates average densities, one can use any version of the annotation volume or any 3D representation of the brain to generate 3D cell atlases, as long as it can match the region ids or the region names, or the parcellations from the AIBS.

Dealing with over 5000 clusters can be daunting. Using the ABC Atlas transcriptomic hierarchy we could combine clusters and their densities into subclasses, supertypes or classes. Since many literature values are available on the classes level, we summed up cluster densities into neuron, inhibitory-, excitatory-, modulatory-, glia (i.e. astrocytes + oligodendrocytes + microglia), astrocyte, oligodendrocyte, microglia densities. This data can be stored in 3D format as *nrrd* files.

Average cell type densities are then easily calculated for the entire annotation hierarchy for validation purposes. For any region in the hierarchy can be broken down to its constituent leaf regions. Cell densities could be summed up and neatly stored in a single dataframe.

**Special regions case**

The pipeline allows for the overwriting of cortical me-types with specialized me-types in specific brain regions (e.g., Cerebellum) when the expected densities for these unique me-types are available. The goal of this customization is to identify optimal groupings of t-types, following their hierarchical classification, so that the densities of the grouped t-types best approximate the expected densities of these specialized me-types. This is achieved by defining the me-type densities as:

$$Densities_{me}=W\cdot Densities_{t}$$

where $W$ represents probabilistic weights that are optimized to minimize the distance between the computed me-type densities and the expected densities for specialized regions.

To implement this, hierarchical clustering of t-types is performed based on their expression profiles, creating clusters that preserve similarities among t-types. These clusters are then used to compute aggregated densities, which are normalized and compared to the custom me-type densities via Euclidean distance. The probabilistic weights $W$ are iteratively adjusted to minimize this distance, ensuring that the derived me-type densities for the specialized regions align closely with the known, expected densities. This method provides a flexible way to integrate region-specific information into the probabilistic map, improving the accuracy of the density estimates for non-cortical brain regions while maintaining consistency with the overall pipeline.

**Morphological types (m-types) list:**

IN_dend_0_ax_0

IN_dend_0_ax_2

IN_dend_0_ax_3

IN_dend_0_ax_4

IN_dend_0_ax_5

IN_dend_0_ax_6

IN_dend_0_ax_8

IN_dend_0_ax_9

IN_dend_1_ax_0

IN_dend_1_ax_1

IN_dend_1_ax_2

IN_dend_1_ax_3

IN_dend_1_ax_4

IN_dend_1_ax_5

IN_dend_1_ax_7

IN_dend_1_ax_8

IN_dend_1_ax_9

IN_dend_2_ax_0

IN_dend_2_ax_2

IN_dend_2_ax_3

IN_dend_2_ax_4

IN_dend_2_ax_5

IN_dend_2_ax_6

IN_dend_2_ax_9

IN_dend_3_ax_0

IN_dend_3_ax_2

IN_dend_3_ax_3

IN_dend_3_ax_5

IN_dend_3_ax_6

IN_dend_3_ax_7

IN_dend_3_ax_8

IN_dend_3_ax_9

IN_dend_4_ax_2

IN_dend_4_ax_3

IN_dend_4_ax_5

IN_dend_4_ax_6

IN_dend_4_ax_8

IN_dend_4_ax_9

IN_dend_5_ax_0

IN_dend_5_ax_3

IN_dend_5_ax_4

IN_dend_5_ax_5

IN_dend_5_ax_6

IN_dend_5_ax_7

IN_dend_5_ax_8

IN_dend_5_ax_9

IN_dend_6_ax_0

IN_dend_6_ax_2

IN_dend_6_ax_3

IN_dend_6_ax_7

IN_dend_6_ax_8

IN_dend_6_ax_9

IN_dend_7_ax_0

IN_dend_7_ax_1

IN_dend_7_ax_2

IN_dend_7_ax_3

IN_dend_7_ax_4

IN_dend_7_ax_7

IN_dend_7_ax_8

IN_dend_8_ax_1

IN_dend_8_ax_7

IN_dend_8_ax_8

IN_dend_8_ax_9

IN_dend_9_ax_0

IN_dend_9_ax_1

IN_dend_9_ax_2

IN_dend_9_ax_3

IN_dend_9_ax_4

IN_dend_9_ax_5

IN_dend_9_ax_8

IN_dend_9_ax_9

PC_dend_0_ax_8

PC_dend_1_ax_7

PC_dend_2_ax_4

PC_dend_2_ax_7

PC_dend_2_ax_8

PC_dend_3_ax_0

PC_dend_3_ax_4

PC_dend_3_ax_6

PC_dend_3_ax_7

PC_dend_3_ax_8

PC_dend_8_ax_4

PC_dend_8_ax_7

PC_dend_8_ax_8

**Electrophysiological types (e-types) list:**

bAC : burst accommodating

bIR : burst irregular

bNAC : burst non-accommodating

bSTUT : burst stuttering

cAC : continuous accommodating

cADpyr : continuous adapting pyramidal neurons

cIR : continuous irregular

cNAC : continuous non-accommodating

cSTUT : continuous stuttering

dNAC : delayed non-accommodating

dSTUT : delayed stuttering

**Morpho-electrophysiological types (me-types) list:**

PC_DEND_0_AX_8|cADpyr

PC_DEND_1_AX_7|cADpyr

PC_DEND_2_AX_4|cADpyr

PC_DEND_2_AX_8|cADpyr

PC_DEND_3_AX_0|cADpyr

PC_DEND_3_AX_4|cADpyr

PC_DEND_3_AX_6|cADpyr

PC_DEND_3_AX_7|cADpyr

PC_DEND_3_AX_8|cADpyr

PC_DEND_8_AX_4|cADpyr

PC_DEND_8_AX_8|cADpyr

IN_DEND_0_AX_0|bAC

IN_DEND_0_AX_0|bIR

IN_DEND_0_AX_0|bNAC

IN_DEND_0_AX_0|bSTUT

IN_DEND_0_AX_0|cAC

IN_DEND_0_AX_0|cIR

IN_DEND_0_AX_0|cNAC

IN_DEND_0_AX_0|cSTUT

IN_DEND_0_AX_0|dNAC

IN_DEND_0_AX_0|dSTUT

IN_DEND_0_AX_3|bAC

IN_DEND_0_AX_3|bIR

IN_DEND_0_AX_3|bNAC

IN_DEND_0_AX_3|bSTUT

IN_DEND_0_AX_3|cAC

IN_DEND_0_AX_3|cIR

IN_DEND_0_AX_3|cNAC

IN_DEND_0_AX_3|cSTUT

IN_DEND_0_AX_3|dNAC

IN_DEND_0_AX_3|dSTUT

IN_DEND_0_AX_4|bAC

IN_DEND_0_AX_4|bIR

IN_DEND_0_AX_4|bNAC

IN_DEND_0_AX_4|bSTUT

IN_DEND_0_AX_4|cAC

IN_DEND_0_AX_4|cIR

IN_DEND_0_AX_4|cNAC

IN_DEND_0_AX_4|cSTUT

IN_DEND_0_AX_4|dSTUT

IN_DEND_0_AX_5|bAC

IN_DEND_0_AX_5|bIR

IN_DEND_0_AX_5|bNAC

IN_DEND_0_AX_5|bSTUT

IN_DEND_0_AX_5|cAC

IN_DEND_0_AX_5|cIR

IN_DEND_0_AX_5|cNAC

IN_DEND_0_AX_5|cSTUT

IN_DEND_0_AX_5|dSTUT

IN_DEND_0_AX_6|bAC

IN_DEND_0_AX_6|bIR

IN_DEND_0_AX_6|bNAC

IN_DEND_0_AX_6|bSTUT

IN_DEND_0_AX_6|cAC

IN_DEND_0_AX_6|cIR

IN_DEND_0_AX_6|cNAC

IN_DEND_0_AX_6|cSTUT

IN_DEND_0_AX_8|bAC

IN_DEND_0_AX_8|bIR

IN_DEND_0_AX_8|bNAC

IN_DEND_0_AX_8|cAC

IN_DEND_0_AX_8|dSTUT

IN_DEND_0_AX_9|bAC

IN_DEND_0_AX_9|bIR

IN_DEND_0_AX_9|bNAC

IN_DEND_0_AX_9|bSTUT

IN_DEND_0_AX_9|cAC

IN_DEND_0_AX_9|cIR

IN_DEND_0_AX_9|cNAC

IN_DEND_0_AX_9|cSTUT

IN_DEND_0_AX_9|dNAC

IN_DEND_0_AX_9|dSTUT

IN_DEND_1_AX_0|bAC

IN_DEND_1_AX_0|bIR

IN_DEND_1_AX_0|bNAC

IN_DEND_1_AX_0|bSTUT

IN_DEND_1_AX_0|cAC

IN_DEND_1_AX_0|cIR

IN_DEND_1_AX_0|cNAC

IN_DEND_1_AX_0|cSTUT

IN_DEND_1_AX_0|dNAC

IN_DEND_1_AX_0|dSTUT

IN_DEND_1_AX_2|bAC

IN_DEND_1_AX_2|bIR

IN_DEND_1_AX_2|bNAC

IN_DEND_1_AX_2|bSTUT

IN_DEND_1_AX_2|cAC

IN_DEND_1_AX_2|cIR

IN_DEND_1_AX_2|cNAC

IN_DEND_1_AX_2|cSTUT

IN_DEND_1_AX_2|dNAC

IN_DEND_1_AX_2|dSTUT

IN_DEND_1_AX_3|bAC

IN_DEND_1_AX_3|bIR

IN_DEND_1_AX_3|bNAC

IN_DEND_1_AX_3|bSTUT

IN_DEND_1_AX_3|cAC

IN_DEND_1_AX_3|cIR

IN_DEND_1_AX_3|cNAC

IN_DEND_1_AX_3|cSTUT

IN_DEND_1_AX_3|dNAC

IN_DEND_1_AX_3|dSTUT

IN_DEND_1_AX_4|bAC

IN_DEND_1_AX_4|bIR

IN_DEND_1_AX_4|bNAC

IN_DEND_1_AX_4|bSTUT

IN_DEND_1_AX_4|cAC

IN_DEND_1_AX_4|cIR

IN_DEND_1_AX_4|cNAC

IN_DEND_1_AX_4|cSTUT

IN_DEND_1_AX_5|bAC

IN_DEND_1_AX_5|bIR

IN_DEND_1_AX_5|bNAC

IN_DEND_1_AX_5|bSTUT

IN_DEND_1_AX_5|cAC

IN_DEND_1_AX_5|cIR

IN_DEND_1_AX_5|cNAC

IN_DEND_1_AX_5|cSTUT

IN_DEND_1_AX_5|dSTUT

IN_DEND_1_AX_7|bAC

IN_DEND_1_AX_7|bIR

IN_DEND_1_AX_7|bNAC

IN_DEND_1_AX_7|bSTUT

IN_DEND_1_AX_7|cAC

IN_DEND_1_AX_7|cIR

IN_DEND_1_AX_7|cNAC

IN_DEND_1_AX_7|cSTUT

IN_DEND_1_AX_8|bAC

IN_DEND_1_AX_8|bIR

IN_DEND_1_AX_8|bNAC

IN_DEND_1_AX_8|bSTUT

IN_DEND_1_AX_8|cAC

IN_DEND_1_AX_8|cIR

IN_DEND_1_AX_8|cNAC

IN_DEND_1_AX_8|cSTUT

IN_DEND_1_AX_9|bAC

IN_DEND_1_AX_9|bIR

IN_DEND_1_AX_9|bNAC

IN_DEND_1_AX_9|bSTUT

IN_DEND_1_AX_9|cAC

IN_DEND_1_AX_9|cIR

IN_DEND_1_AX_9|cNAC

IN_DEND_1_AX_9|cSTUT

IN_DEND_1_AX_9|dNAC

IN_DEND_1_AX_9|dSTUT

IN_DEND_2_AX_2|bAC

IN_DEND_2_AX_2|bIR

IN_DEND_2_AX_2|bNAC

IN_DEND_2_AX_2|bSTUT

IN_DEND_2_AX_2|cAC

IN_DEND_2_AX_2|cIR

IN_DEND_2_AX_2|cNAC

IN_DEND_2_AX_2|cSTUT

IN_DEND_2_AX_2|dNAC

IN_DEND_2_AX_2|dSTUT

IN_DEND_2_AX_3|bAC

IN_DEND_2_AX_3|bIR

IN_DEND_2_AX_3|bNAC

IN_DEND_2_AX_3|bSTUT

IN_DEND_2_AX_3|cAC

IN_DEND_2_AX_3|cIR

IN_DEND_2_AX_3|cNAC

IN_DEND_2_AX_3|cSTUT

IN_DEND_2_AX_3|dNAC

IN_DEND_2_AX_3|dSTUT

IN_DEND_3_AX_2|bAC

IN_DEND_3_AX_2|bIR

IN_DEND_3_AX_2|bNAC

IN_DEND_3_AX_2|bSTUT

IN_DEND_3_AX_2|cAC

IN_DEND_3_AX_2|cIR

IN_DEND_3_AX_2|cNAC

IN_DEND_3_AX_2|cSTUT

IN_DEND_3_AX_2|dNAC

IN_DEND_3_AX_2|dSTUT

IN_DEND_3_AX_3|bAC

IN_DEND_3_AX_3|bIR

IN_DEND_3_AX_3|bNAC

IN_DEND_3_AX_3|bSTUT

IN_DEND_3_AX_3|cAC

IN_DEND_3_AX_3|cIR

IN_DEND_3_AX_3|cNAC

IN_DEND_3_AX_3|cSTUT

IN_DEND_3_AX_3|dNAC

IN_DEND_3_AX_3|dSTUT

IN_DEND_3_AX_5|bAC

IN_DEND_3_AX_5|bIR

IN_DEND_3_AX_5|bNAC

IN_DEND_3_AX_5|bSTUT

IN_DEND_3_AX_5|cAC

IN_DEND_3_AX_5|cIR

IN_DEND_3_AX_5|cNAC

IN_DEND_3_AX_5|cSTUT

IN_DEND_3_AX_7|bAC

IN_DEND_3_AX_7|bIR

IN_DEND_3_AX_7|bNAC

IN_DEND_3_AX_7|cAC

IN_DEND_3_AX_7|dSTUT

IN_DEND_4_AX_2|bAC

IN_DEND_4_AX_2|bIR

IN_DEND_4_AX_2|bNAC

IN_DEND_4_AX_2|bSTUT

IN_DEND_4_AX_2|cAC

IN_DEND_4_AX_2|cIR

IN_DEND_4_AX_2|cNAC

IN_DEND_4_AX_2|cSTUT

IN_DEND_4_AX_2|dNAC

IN_DEND_4_AX_2|dSTUT

IN_DEND_4_AX_3|bAC

IN_DEND_4_AX_3|bIR

IN_DEND_4_AX_3|bNAC

IN_DEND_4_AX_3|bSTUT

IN_DEND_4_AX_3|cAC

IN_DEND_4_AX_3|cIR

IN_DEND_4_AX_3|cNAC

IN_DEND_4_AX_3|cSTUT

IN_DEND_4_AX_3|dNAC

IN_DEND_4_AX_3|dSTUT

IN_DEND_4_AX_5|bAC

IN_DEND_4_AX_5|bIR

IN_DEND_4_AX_5|bNAC

IN_DEND_4_AX_5|bSTUT

IN_DEND_4_AX_5|cAC

IN_DEND_4_AX_5|cIR

IN_DEND_4_AX_5|cNAC

IN_DEND_4_AX_5|cSTUT

IN_DEND_4_AX_5|dNAC

IN_DEND_4_AX_5|dSTUT

IN_DEND_4_AX_6|bAC

IN_DEND_4_AX_6|bIR

IN_DEND_4_AX_6|bNAC

IN_DEND_4_AX_6|bSTUT

IN_DEND_4_AX_6|cAC

IN_DEND_4_AX_6|cIR

IN_DEND_4_AX_6|cNAC

IN_DEND_4_AX_6|cSTUT

IN_DEND_4_AX_6|dNAC

IN_DEND_4_AX_6|dSTUT

IN_DEND_4_AX_8|bAC

IN_DEND_4_AX_8|bIR

IN_DEND_4_AX_8|bNAC

IN_DEND_4_AX_8|bSTUT

IN_DEND_4_AX_8|cAC

IN_DEND_4_AX_8|cIR

IN_DEND_4_AX_8|cNAC

IN_DEND_4_AX_8|cSTUT

IN_DEND_4_AX_8|dNAC

IN_DEND_4_AX_8|dSTUT

IN_DEND_4_AX_9|bAC

IN_DEND_4_AX_9|bIR

IN_DEND_4_AX_9|bNAC

IN_DEND_4_AX_9|bSTUT

IN_DEND_4_AX_9|cAC

IN_DEND_4_AX_9|cIR

IN_DEND_4_AX_9|cNAC

IN_DEND_4_AX_9|cSTUT

IN_DEND_4_AX_9|dNAC

IN_DEND_4_AX_9|dSTUT

IN_DEND_5_AX_0|bAC

IN_DEND_5_AX_0|bIR

IN_DEND_5_AX_0|bNAC

IN_DEND_5_AX_0|bSTUT

IN_DEND_5_AX_0|cAC

IN_DEND_5_AX_0|cIR

IN_DEND_5_AX_0|cNAC

IN_DEND_5_AX_0|cSTUT

IN_DEND_5_AX_0|dNAC

IN_DEND_5_AX_0|dSTUT

IN_DEND_5_AX_3|bAC

IN_DEND_5_AX_3|bIR

IN_DEND_5_AX_3|bNAC

IN_DEND_5_AX_3|bSTUT

IN_DEND_5_AX_3|cAC

IN_DEND_5_AX_3|cIR

IN_DEND_5_AX_3|cNAC

IN_DEND_5_AX_3|cSTUT

IN_DEND_5_AX_3|dNAC

IN_DEND_5_AX_3|dSTUT

IN_DEND_5_AX_4|bAC

IN_DEND_5_AX_4|bIR

IN_DEND_5_AX_4|bNAC

IN_DEND_5_AX_4|cAC

IN_DEND_5_AX_4|dSTUT

IN_DEND_5_AX_5|bAC

IN_DEND_5_AX_5|bIR

IN_DEND_5_AX_5|bNAC

IN_DEND_5_AX_5|bSTUT

IN_DEND_5_AX_5|cAC

IN_DEND_5_AX_5|cIR

IN_DEND_5_AX_5|cNAC

IN_DEND_5_AX_5|cSTUT

IN_DEND_5_AX_5|dNAC

IN_DEND_5_AX_5|dSTUT

IN_DEND_5_AX_6|bAC

IN_DEND_5_AX_6|bIR

IN_DEND_5_AX_6|bNAC

IN_DEND_5_AX_6|bSTUT

IN_DEND_5_AX_6|cAC

IN_DEND_5_AX_6|cIR

IN_DEND_5_AX_6|cNAC

IN_DEND_5_AX_6|cSTUT

IN_DEND_5_AX_6|dSTUT

IN_DEND_5_AX_7|bAC

IN_DEND_5_AX_7|bIR

IN_DEND_5_AX_7|bNAC

IN_DEND_5_AX_7|cAC

IN_DEND_5_AX_7|dSTUT

IN_DEND_5_AX_8|bAC

IN_DEND_5_AX_8|bIR

IN_DEND_5_AX_8|bNAC

IN_DEND_5_AX_8|bSTUT

IN_DEND_5_AX_8|cAC

IN_DEND_5_AX_8|cIR

IN_DEND_5_AX_8|cNAC

IN_DEND_5_AX_8|cSTUT

IN_DEND_5_AX_8|dNAC

IN_DEND_5_AX_8|dSTUT

IN_DEND_5_AX_9|bAC

IN_DEND_5_AX_9|bIR

IN_DEND_5_AX_9|bNAC

IN_DEND_5_AX_9|bSTUT

IN_DEND_5_AX_9|cAC

IN_DEND_5_AX_9|cIR

IN_DEND_5_AX_9|cNAC

IN_DEND_5_AX_9|cSTUT

IN_DEND_5_AX_9|dNAC

IN_DEND_5_AX_9|dSTUT

IN_DEND_6_AX_0|bAC

IN_DEND_6_AX_0|bIR

IN_DEND_6_AX_0|bNAC

IN_DEND_6_AX_0|bSTUT

IN_DEND_6_AX_0|cAC

IN_DEND_6_AX_0|cIR

IN_DEND_6_AX_0|cNAC

IN_DEND_6_AX_0|cSTUT

IN_DEND_6_AX_0|dNAC

IN_DEND_6_AX_0|dSTUT

IN_DEND_6_AX_3|bAC

IN_DEND_6_AX_3|bIR

IN_DEND_6_AX_3|bNAC

IN_DEND_6_AX_3|bSTUT

IN_DEND_6_AX_3|cAC

IN_DEND_6_AX_3|cIR

IN_DEND_6_AX_3|cNAC

IN_DEND_6_AX_3|cSTUT

IN_DEND_6_AX_3|dNAC

IN_DEND_6_AX_3|dSTUT

IN_DEND_6_AX_8|bAC

IN_DEND_6_AX_8|bIR

IN_DEND_6_AX_8|bNAC

IN_DEND_6_AX_8|bSTUT

IN_DEND_6_AX_8|cAC

IN_DEND_6_AX_8|cIR

IN_DEND_6_AX_8|cNAC

IN_DEND_6_AX_8|cSTUT

IN_DEND_6_AX_8|dNAC

IN_DEND_6_AX_8|dSTUT

IN_DEND_6_AX_9|bAC

IN_DEND_6_AX_9|bIR

IN_DEND_6_AX_9|bNAC

IN_DEND_6_AX_9|bSTUT

IN_DEND_6_AX_9|cAC

IN_DEND_6_AX_9|cIR

IN_DEND_6_AX_9|cNAC

IN_DEND_6_AX_9|cSTUT

IN_DEND_6_AX_9|dSTUT

IN_DEND_7_AX_0|bAC

IN_DEND_7_AX_0|bIR

IN_DEND_7_AX_0|bNAC

IN_DEND_7_AX_0|bSTUT

IN_DEND_7_AX_0|cAC

IN_DEND_7_AX_0|cIR

IN_DEND_7_AX_0|cNAC

IN_DEND_7_AX_0|cSTUT

IN_DEND_7_AX_0|dNAC

IN_DEND_7_AX_0|dSTUT

IN_DEND_7_AX_2|bAC

IN_DEND_7_AX_2|bIR

IN_DEND_7_AX_2|bNAC

IN_DEND_7_AX_2|bSTUT

IN_DEND_7_AX_2|cAC

IN_DEND_7_AX_2|cIR

IN_DEND_7_AX_2|cNAC

IN_DEND_7_AX_2|cSTUT

IN_DEND_7_AX_2|dNAC

IN_DEND_7_AX_2|dSTUT

IN_DEND_7_AX_3|bAC

IN_DEND_7_AX_3|bIR

IN_DEND_7_AX_3|bNAC

IN_DEND_7_AX_3|bSTUT

IN_DEND_7_AX_3|cAC

IN_DEND_7_AX_3|cIR

IN_DEND_7_AX_3|cNAC

IN_DEND_7_AX_3|cSTUT

IN_DEND_7_AX_3|dNAC

IN_DEND_7_AX_3|dSTUT

IN_DEND_7_AX_8|bAC

IN_DEND_7_AX_8|bIR

IN_DEND_7_AX_8|bNAC

IN_DEND_7_AX_8|bSTUT

IN_DEND_7_AX_8|cAC

IN_DEND_7_AX_8|cIR

IN_DEND_7_AX_8|cNAC

IN_DEND_7_AX_8|cSTUT

IN_DEND_7_AX_8|dNAC

IN_DEND_7_AX_8|dSTUT

IN_DEND_8_AX_8|bAC

IN_DEND_8_AX_8|bIR

IN_DEND_8_AX_8|bNAC

IN_DEND_8_AX_8|cAC

IN_DEND_8_AX_8|dSTUT

IN_DEND_8_AX_9|bAC

IN_DEND_8_AX_9|bIR

IN_DEND_8_AX_9|bNAC

IN_DEND_8_AX_9|bSTUT

IN_DEND_8_AX_9|cAC

IN_DEND_8_AX_9|cIR

IN_DEND_8_AX_9|cNAC

IN_DEND_8_AX_9|cSTUT

IN_DEND_9_AX_1|bAC

IN_DEND_9_AX_1|bIR

IN_DEND_9_AX_1|bNAC

IN_DEND_9_AX_1|bSTUT

IN_DEND_9_AX_1|cAC

IN_DEND_9_AX_1|cIR

IN_DEND_9_AX_1|cNAC

IN_DEND_9_AX_1|cSTUT

IN_DEND_9_AX_1|dSTUT

IN_DEND_9_AX_2|bAC

IN_DEND_9_AX_2|bIR

IN_DEND_9_AX_2|bNAC

IN_DEND_9_AX_2|bSTUT

IN_DEND_9_AX_2|cAC

IN_DEND_9_AX_2|cIR

IN_DEND_9_AX_2|cNAC

IN_DEND_9_AX_2|cSTUT

IN_DEND_9_AX_2|dNAC

IN_DEND_9_AX_2|dSTUT

IN_DEND_9_AX_5|bAC

IN_DEND_9_AX_5|bIR

IN_DEND_9_AX_5|bNAC

IN_DEND_9_AX_5|bSTUT

IN_DEND_9_AX_5|cAC

IN_DEND_9_AX_5|cIR

IN_DEND_9_AX_5|cNAC

IN_DEND_9_AX_5|cSTUT

IN_DEND_9_AX_5|dNAC

IN_DEND_9_AX_5|dSTUT

IN_DEND_9_AX_8|bAC

IN_DEND_9_AX_8|bIR

IN_DEND_9_AX_8|bNAC

IN_DEND_9_AX_8|bSTUT

IN_DEND_9_AX_8|cAC

IN_DEND_9_AX_8|cIR

IN_DEND_9_AX_8|cNAC

IN_DEND_9_AX_8|cSTUT

IN_DEND_9_AX_8|dNAC

IN_DEND_9_AX_8|dSTUT

**Color map for me-types in Figure 6:**


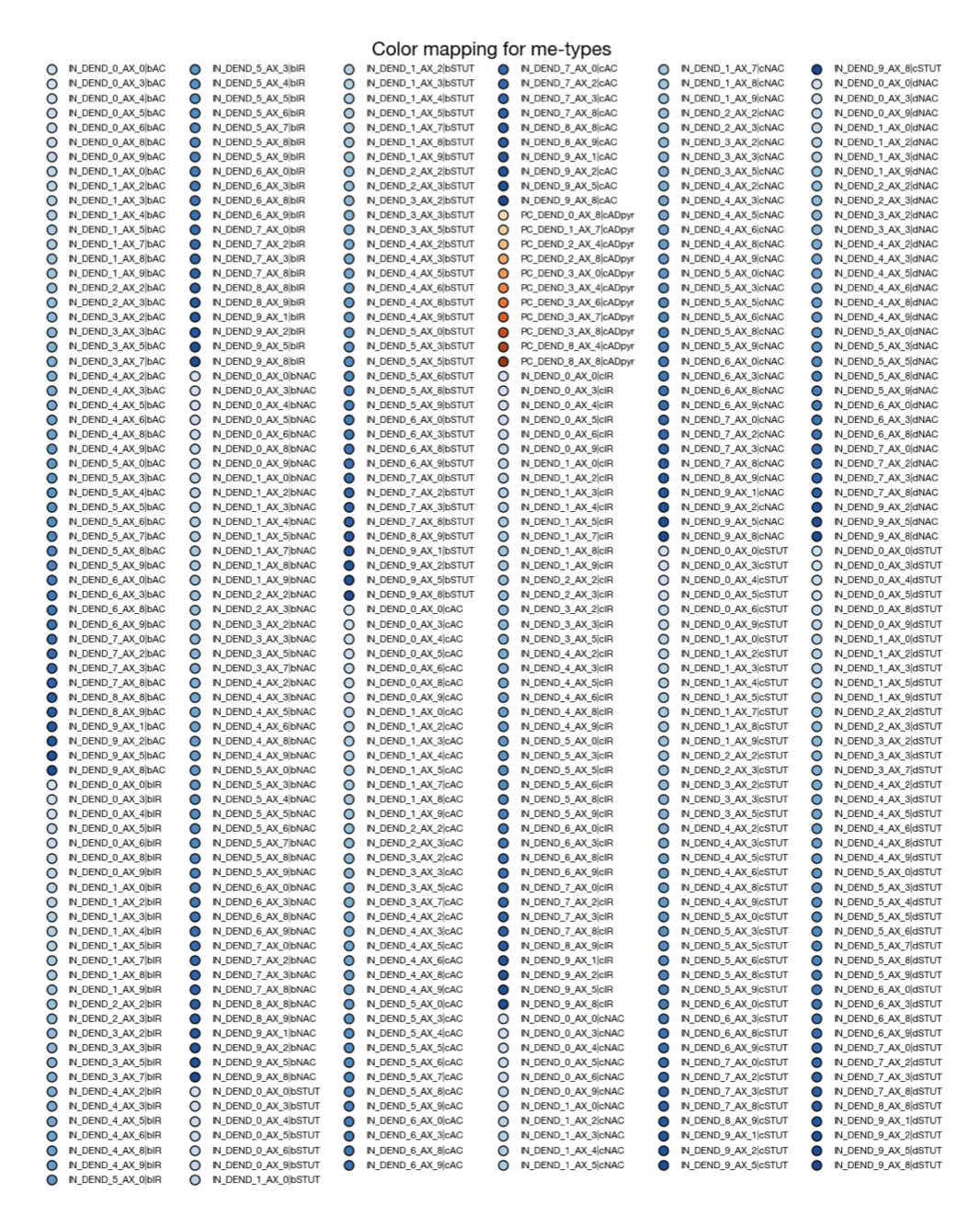

Supplement: S1 Text — (DOCX) [file pcbi.1014106.s006.docx]
