# Supplementary figures and images for: A multimodal spatial atlas of transcriptomic, morphological, and electrophysiological cell type densities in the mouse brain
Source: PLoS Comput Biol. 2026 Mar 24;22(3):e1014106. doi: 10.1371/journal.pcbi.1014106 (PMC13120702; doi:10.1371/journal.pcbi.1014106)

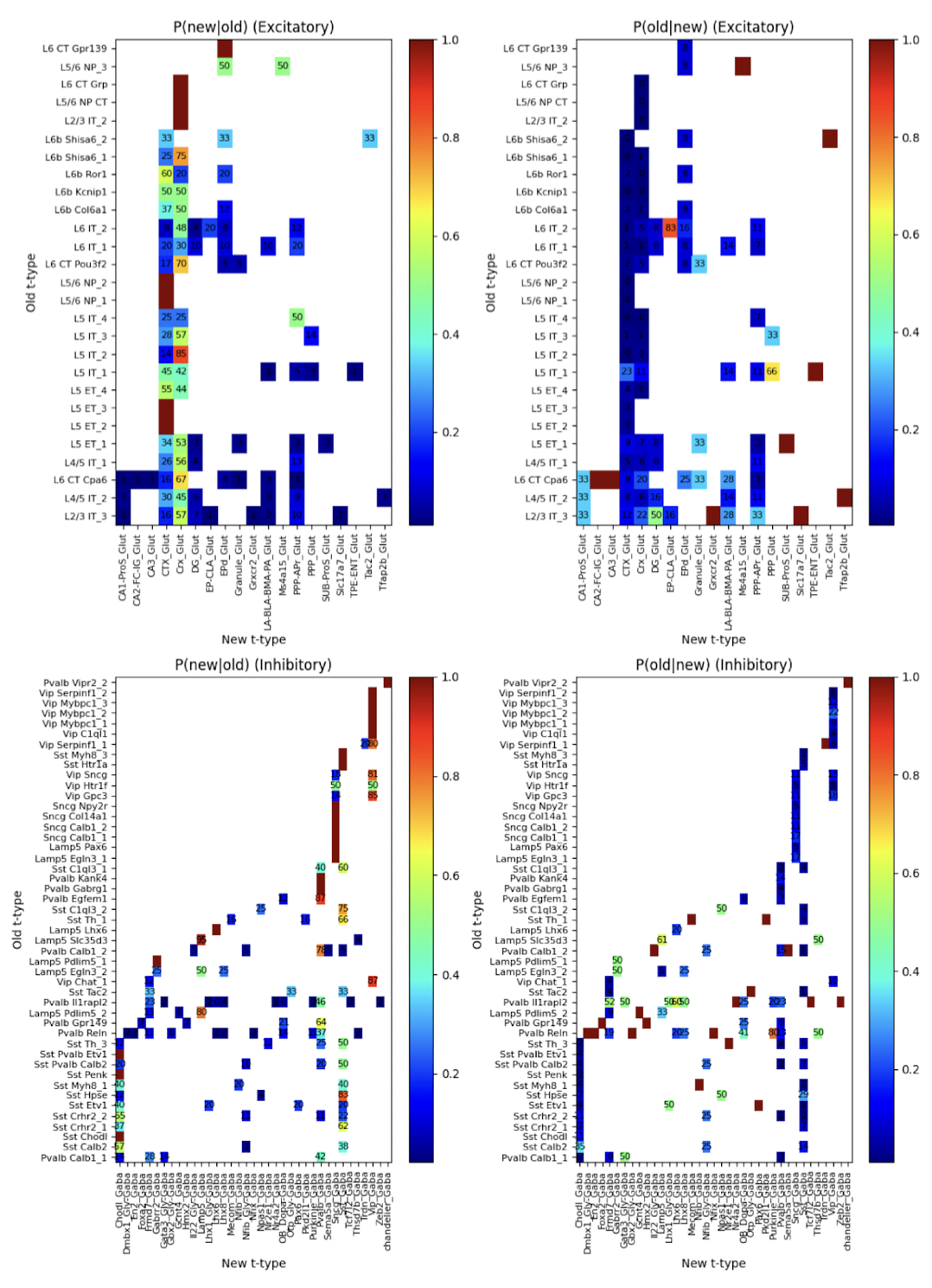

Supplement: S1 Fig — Probability maps highlighting the t-type alignment between native labels (given by the patch-seq datasets) with the assigned reference t-types [54]. Excitatory t-types are on the top row, inhibitory on the bottom. Probabilities of having a t-type from [8] given a native label are on the left, while probabilities of observing a native label given a t-type from [8] are shown on the right. (TIFF) [file pcbi.1014106.s007.tiff]

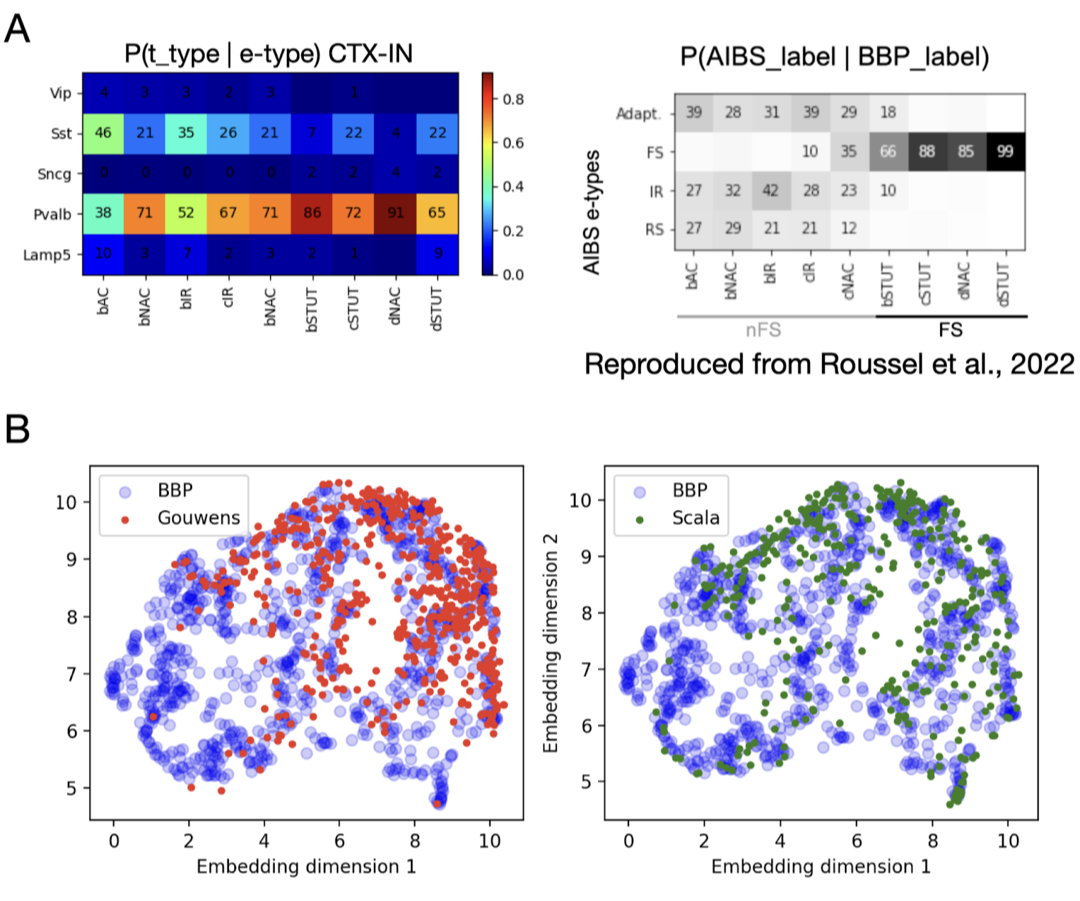

Supplement: S2 Fig — A. Probabilities of observing a canonical e-type given a t-type from [8] in the patch-seq dataset from [30] (left). Probabilities of observing e-types as defined by [26] given canonical e-types (right, reproduced from [48]). bSTUT, cSTUT, dNAC and dSTUT mapped preferentially to fast spiking cells (FS) while all the other mapped mostly to non fast spiking neurons (nFS) B. Coverage of the [29] dataset (left) and the patch-seq dataset from [30] of the embedding of the canonical morphological space are defined by the reference morphological dataset (labeled as “BBP”). (TIFF) [file pcbi.1014106.s008.tiff]

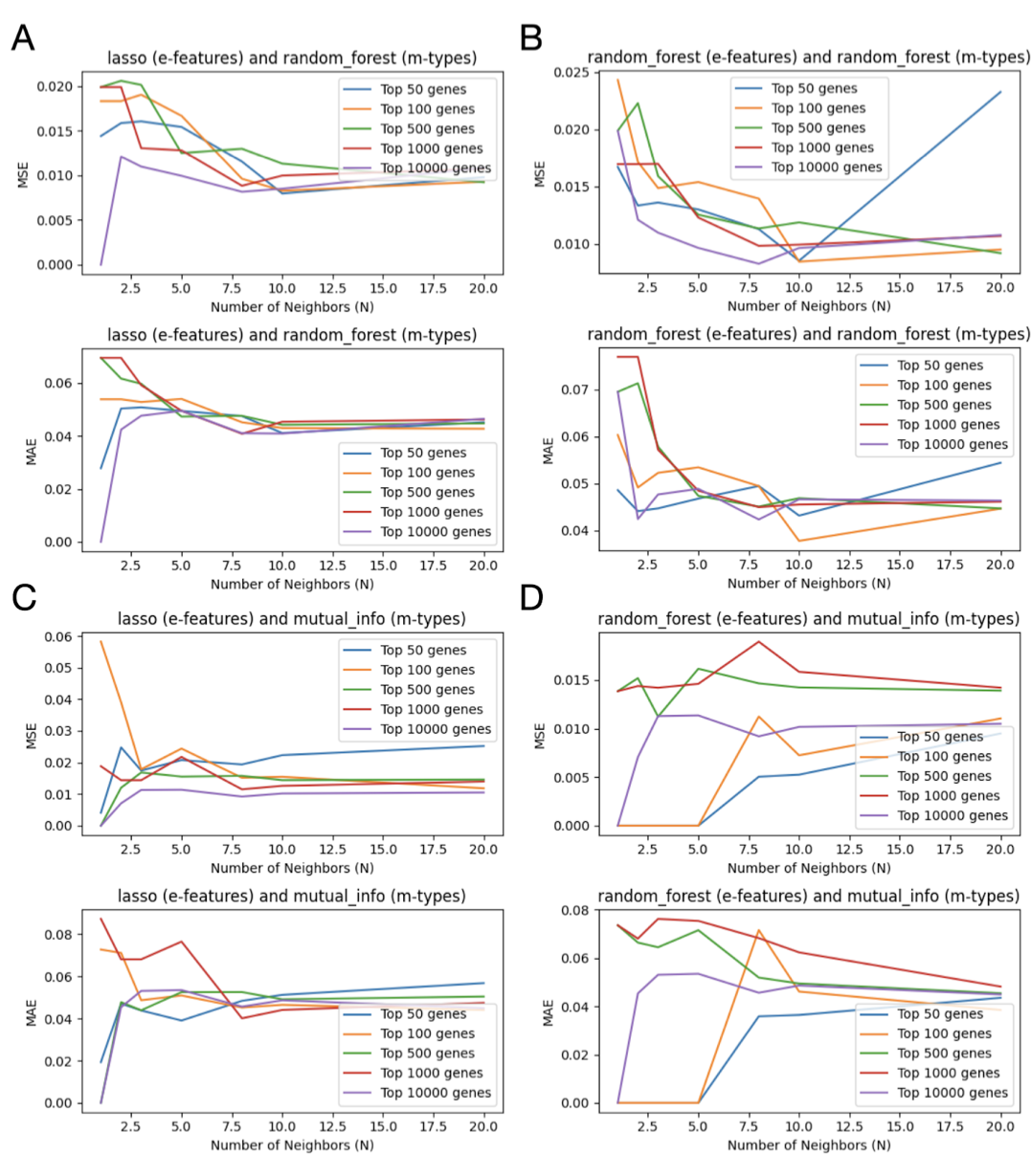

Supplement: S3 Fig — (Ngenes, Nneighbors, Clustering algorithm). Mean squared error (MSE, top) and mean absolute error (MAE, bottom) as a function of Nneighbors for Ngenes=50,100,500,1000 and 10000 and for multiple machine learning algorithms combination for e-features space and m-types space gene selection for the 10-fold cross-validation process. Several combinations were tried: lasso and random forest (A), random forest and random forest (B), lasso and mutual info (C), random forest and mutual info (D). (TIFF) [file pcbi.1014106.s009.tiff]

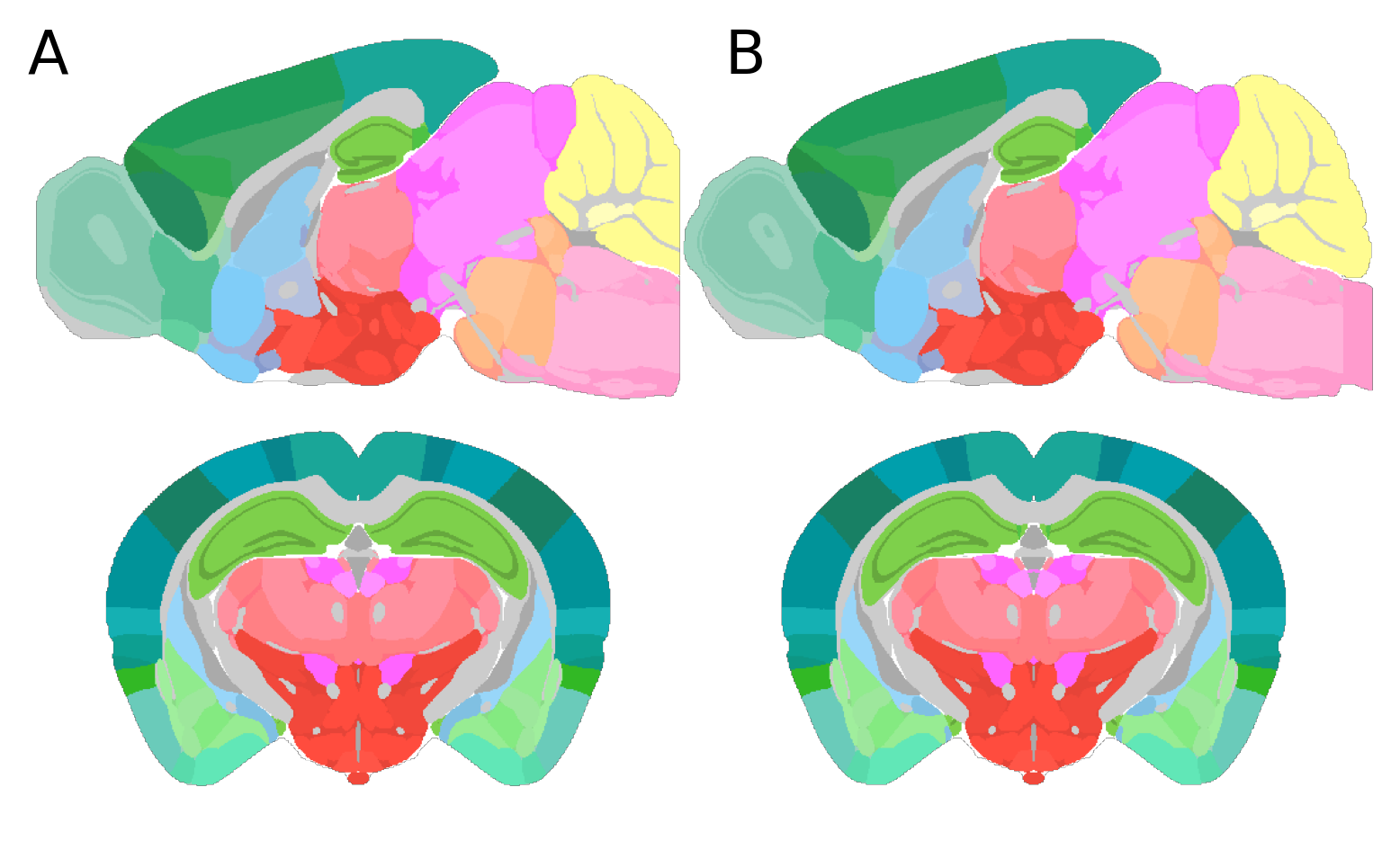

Supplement: S4 Fig — A. Sagittal (y = 200) and coronal (x = 315) sections of the CCFv3 annotation volume (from the AIBS (x = 300) and the B. extended CCFv3 annotation volume from the literature [33]. The legend annotation colors match those in the Allen Institute reference atlas. Resolution: 25 μm3 voxel size. (TIFF) [file pcbi.1014106.s010.tiff]

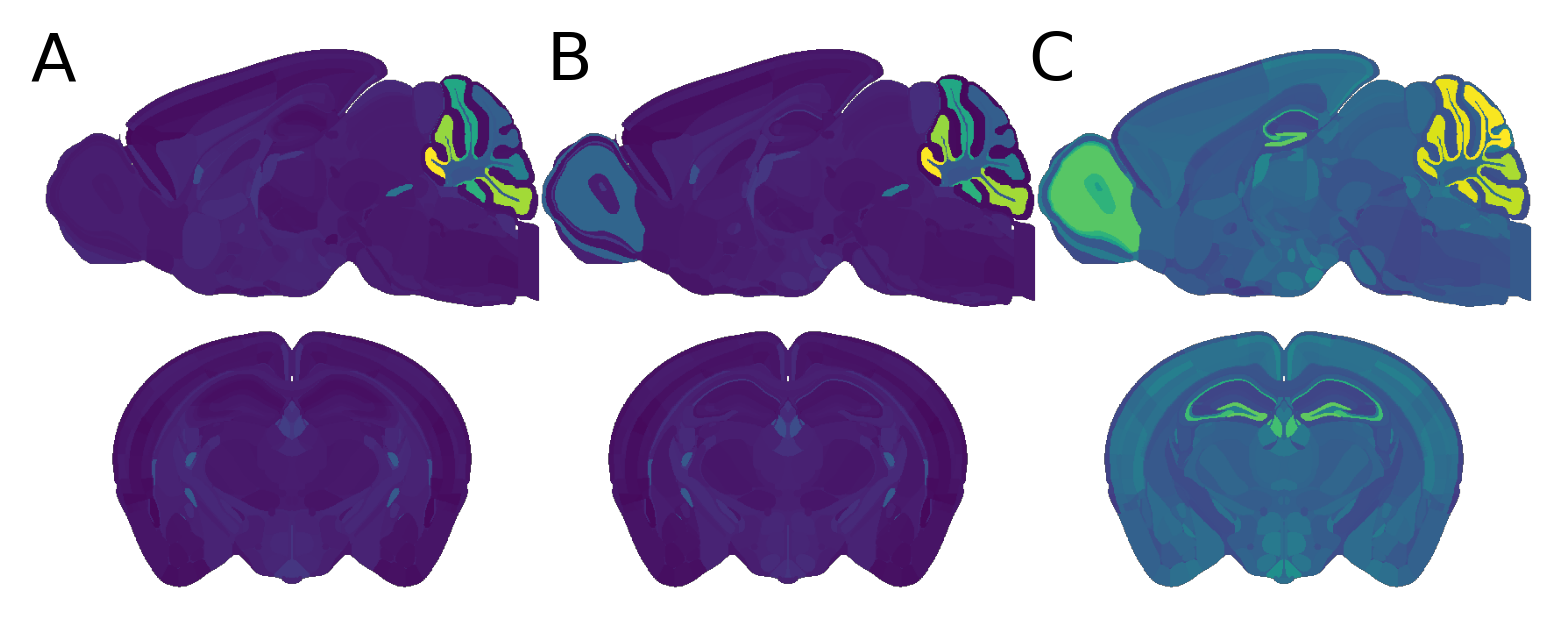

Supplement: S5 Fig — Sagittal (y = 200) and coronal (x = 300) sections of scaled density volumes including all cell types: A. Scaled to match total cell numbers reported in [5]; B. Scaled to match total cell numbers from [5] with transplant adjustment; C. Regional total cell type densities scaled to reflect differences in Nissl staining intensity. Resolution: 25 μm3 voxel size. (TIFF) [file pcbi.1014106.s011.tiff]

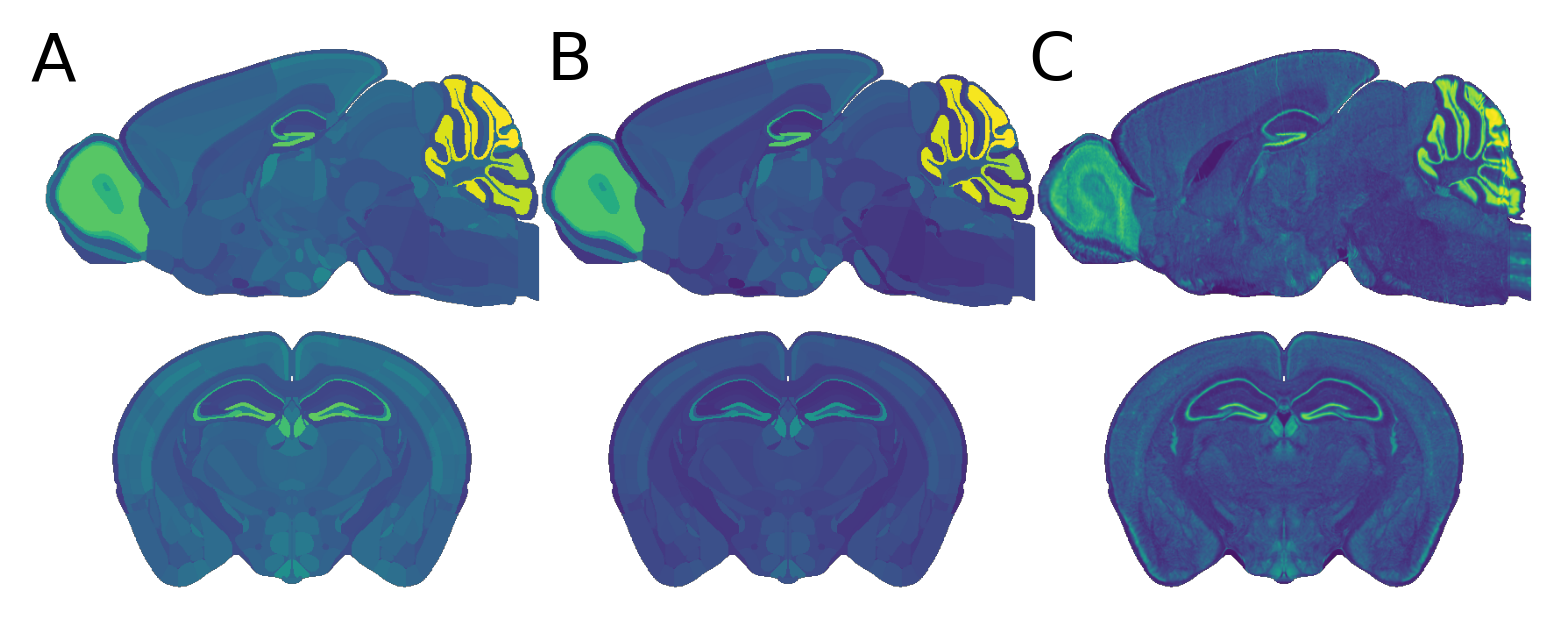

Supplement: S6 Fig — Sagittal (y = 200) and coronal (x = 300) sections of scaled density volumes (total cell): A. All regional total cell densities are scaled to reflect differences in Nissl intensity, scaled with the minimum Nissl intensity; B. All regional total cell densities are scaled to reflect differences in Nissl intensity, scaled with the maximum Nissl intensity equals 4 million cells/mm3; C. Nissl granularity was added to A to emulate variance within each region. Resolution: 25 μm3 voxel size. (TIFF) [file pcbi.1014106.s012.tiff]

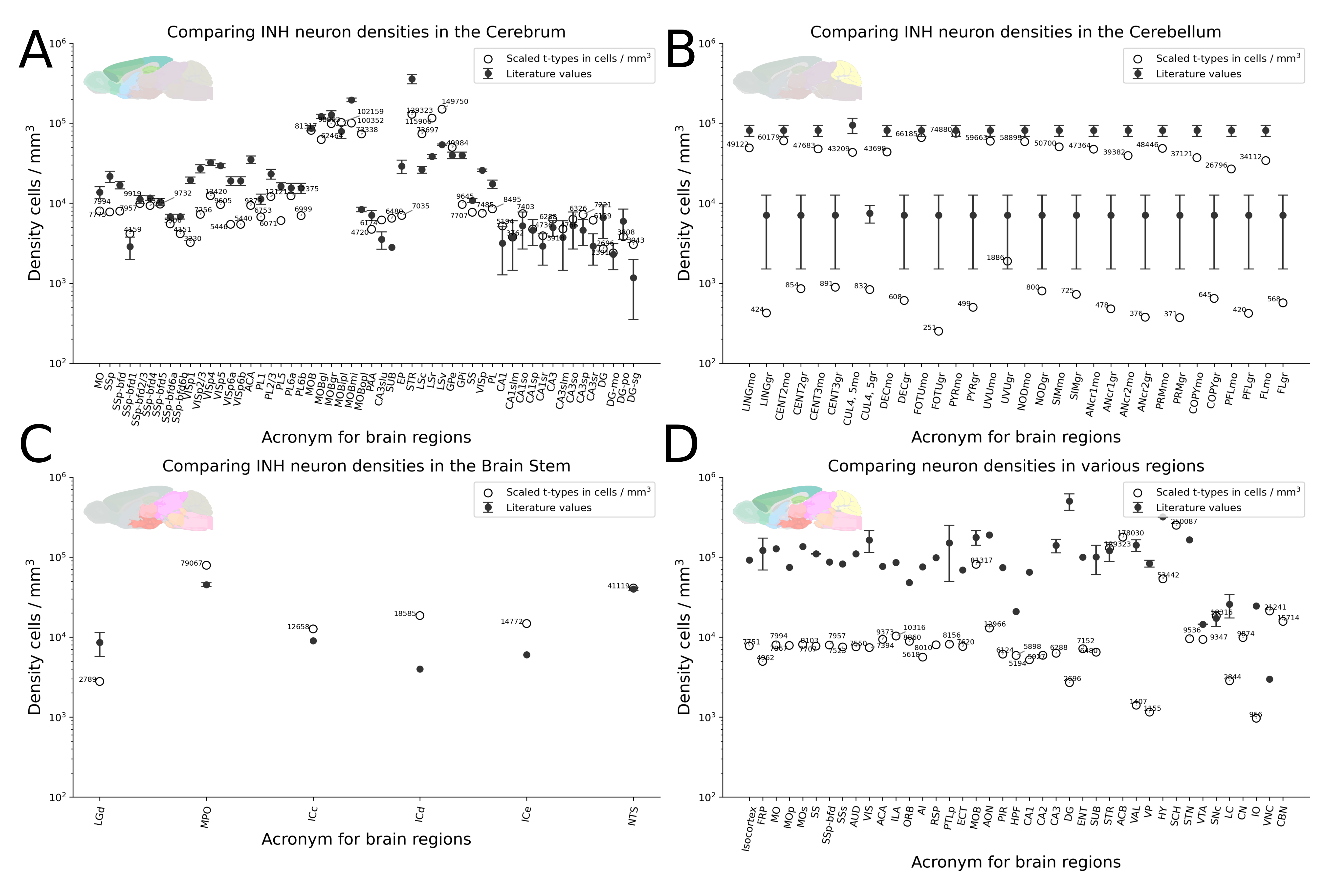

Supplement: S7 Fig — A. We extracted inhibitory neuron densities from the cerebrum from the scaled atlas and compared them with inhibitory neuron numbers from the literature [7,38]. B. Inhibitory densities extracted from the cerebellum, and C. the brain stem. Comparison of total neuron density values from the scaled atlas with values from the literature [7,38]. Insets in the top-left corners show sampled areas, colored according to the annotation scheme used in the AIBS reference atlas. For regions with multiple reported literature values, we depict the variability using error bars. Acronyms are listed in S2 Table. (TIFF) [file pcbi.1014106.s013.tiff]

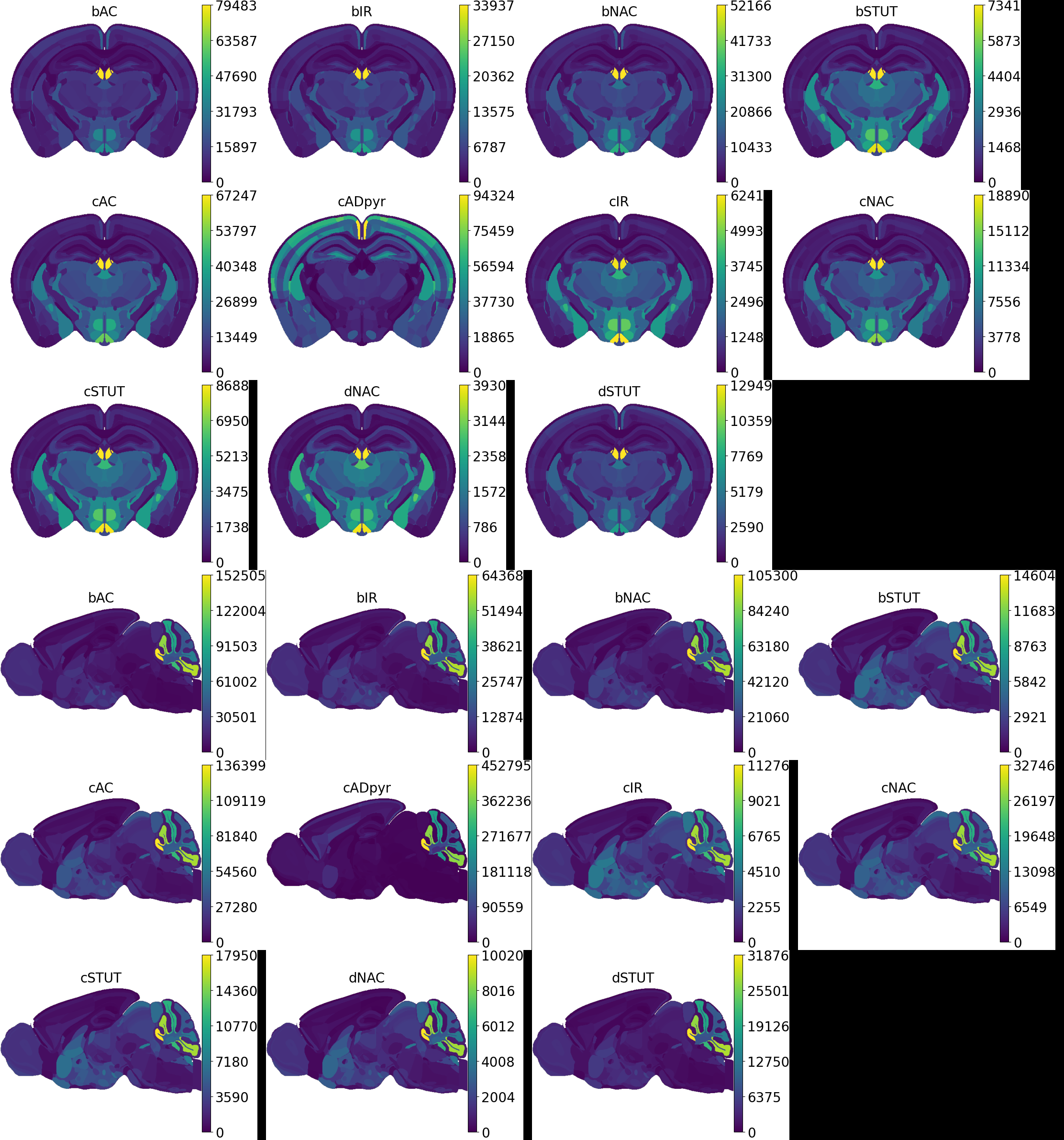

Supplement: S8 Fig — Coronal (x = 300) and sagittal (y = 200) sections of scaled density volumes of all 11 e-types. Density values were projected in the extended and improved CCFv3 annotation volume. Resolution: 25 μm3 voxel size. Colorbars show the number of cells / mm3 (rounded) for every panel. Abbreviations: cADpyr: continuous adapting pyramidal neuron (excitatory), bAC: bursting accommodating (inhibitory), bIR: bursting irregular spiking (inhibitory), bNAC: bursting non-adapting (inhibitory), bSTUT: bursting stuttering (inhibitory), cAC: continuous adapting (inhibitory), cIR: continuous irregular spiking (inhibitory), cNAC: continuous non-adapting (inhibitory), cSTUT: continuous stuttering (inhibitory), dNAC: delayed non-accommodating (inhibitory), dSTUT: delayed stuttering neuron (inhibitory) (TIFF) [file pcbi.1014106.s014.tiff]
